# Psychological interventions for mental health in adults with multiple long-term conditions: A scoping review and evolution of the evidence base

**DOI:** 10.1371/journal.pone.0355366

**Published:** 2026-10-13

**Authors:** Masha Remskar, Colin J. Greaves, Carolyn A. Chew-Graham, Afroditi Stathi, Gerhard Andersson, Tom Kingstone, Simon Hayhoe, Jeffrey D. Lambert

**Affiliations:** 1 Department for Health, University of Bath, Bath, United Kingdom; 2 College of Health Solutions, Arizona State University, Tempe, Arizona, United States of America; 3 School of Sport, Exercise and Rehabilitation Sciences, University of Birmingham, Birmingham, United Kingdom; 4 School of Medicine, Keele University, Keele, United Kingdom; 5 Research and Innovation Department, Midlands Partnership University NHS Foundation Trust, Stafford, United Kingdom; 6 Department of Behavioural Sciences and Learning, Linköping University, Linköping, Sweden; 7 Department of Biomedical and Clinical Sciences, Linköping University, Linköping, Sweden; 8 Department of Clinical Neuroscience, Karolinska Institute, Solna, Sweden; 9 Lusófona University HEI-Lab: Digital Human-Environment Interaction Labs, Lisbon, Portugal; 10 School of Education, University of Exeter, Exeter, United Kingdom; University of Education Winneba Faculty of Science Education, GHANA

## Abstract

**Background:**

Managing multiple long-term conditions (MLTCs) within individuals is a key global public health priority. Psychological interventions are effective in improving mental health outcomes in single-condition populations, yet the evidence for adults with MLTCs remains scarce. This scoping review aimed to map existing evidence on psychological interventions for people with MLTCs, highlight literature gaps and offer recommendations for future research, practice, and policy.

**Methods:**

The pre-registered review follows Arksey & O’Malley’s (2005) framework and is reported in line with the PRISMA-ScR guidelines. We searched six databases (PubMed, Embase, Scopus, Cochrane, PsycNET, Web of Science) up to January 2025, including reference screening and a grey literature search. After screening 124 full texts, 54 studies were included. Eligible studies evaluated any psychological intervention in adults with two or more long-term health conditions and included a validated psychological health outcome.

**Results:**

Existing research primarily focusses on a handful of chronic conditions (diabetes, cardiovascular disease, chronic pain), often alongside comorbid depression. Cognitive-behavioural therapy (CBT) and behavioural activation (BA) are the most frequently evaluated interventions, generally demonstrating positive effects on mental health and acceptability to patients. The evidence base has progressed from early-stage development and feasibility studies to effectiveness trials, though economic evaluations and implementation research are still scarce. Notable gaps include limited research on transdiagnostic approaches, underserved populations and studies involving healthcare providers and carers. Most studies are concentrated in Western countries, highlighting a need for broader geographic representation, particularly in the global South.

**Conclusions:**

Psychological interventions such as CBT and BA show promise for individuals with MLTCs, but significant gaps remain. Future research should i) prioritise inclusive, transdiagnostic approaches to MLTC management, ii) expand geographic diversity, iii) use data-driven approaches to identify and intervene on common MLTC clusters and iv) incorporate health economic and implementation considerations to support more effective and equitable mental health care for those living with MLTCs.

## Introduction

The co-occurrence of multiple long-term conditions (MLTCs), sometimes referred to as multimorbidity, has been described as “the most common chronic condition” globally [1], affecting roughly one third of adults [2]. Factors such as an ageing population, lifestyle-related risks and advances in medical care that extend life expectancy contribute to this growing prevalence [3]. For individuals, living with MLTCs often means navigating complex healthcare needs, managing multiple treatments and coping with the psychological and physical effects of chronic illness. From a healthcare system perspective, multimorbidity significantly increases resource use: Patients with MLTCs account for an estimated 78% of all primary care consultations in England [4] and healthcare costs rise sharply with each additional condition [5].

The presence of MLTCs not only complicates physical health management but also profoundly impacts mental health. Individuals with MLTCs face double the risk for depression compared to those with a single LTC and triple the risk compared to healthy individuals [6]. Anxiety and psychological distress display similar risk profiles [7]. Moreover, living with a mental health condition is itself a substantial risk factor for developing multimorbidity: Depression and anxiety increase the risk of developing physical conditions nearly two-fold [8]. This interplay between physical and mental health conditions can exacerbate the overall disease burden, leading to poorer health outcomes and a diminished quality of life [9]. Furthermore, mental health difficulties can negatively influence disease progression, adherence to treatment and the ability to engage in activities of daily living [10].

Given the bidirectional relationship between physical and mental health decline in individuals with MLTCs, there is a pressing need for effective psychological interventions [3]. These interventions are designed to reduce psychological distress and improve mental health outcomes, which in turn support physical health improvements [11]. Several psychological approaches have strong evidence of efficacy in populations with a single LTC: Cognitive-behavioural therapy (CBT) is considered the gold standard approach for individuals with depression [12] and has shown effectiveness for individuals with physical LTCs [13]. Other evidence-based approaches include mindfulness-based interventions (MBIs) and behavioural activation (BA) [14,15]. Despite a strong evidence base, the effectiveness of psychological interventions in people with MLTCs remains comparatively underexplored and warrants further study [1].

Research on MLTCs is in relatively early stages and facing several challenges. Firstly, a historical focus on single conditions in research and clinical practice has led to a legacy of assuming that single-condition insights are merely additive [16]. This perspective overlooks the complexity of living with MLTCs, where interactions between conditions’ symptoms, effects and treatments can lead to unexpected outcomes or an added burden. Secondly, the evidence base for interventions specifically targeting individuals with MLTCs is underdeveloped considering its rising prevalence and impact, [2] reflecting the relatively recent recognition of the complexity it entails (both experientially and in terms of treatment needs) [10,17] and uptick in dedicated research funding [18,19]. Thirdly, the literature is fragmented into groupings of conditions based on incidental or funding-mandated foci (e.g., funding calls for interventions targeting a specific class of conditions) [20], making a coherent overview difficult. Finally, the accumulation of knowledge on MLTC treatment is further complicated by variation in the definition of a long-term health condition (i.e., whether to include only physical conditions or also chronic mental health conditions) [21]. In this review, we adhere to the UK Academy of Medical Sciences’ definition, where each of the co-occurring conditions is either i) a physical non-communicable disease of long duration, ii) a mental health condition of long duration, or iii) an infectious disease of long duration [21]. As a result of these four challenges, existing reviews synthesise intervention literature for specific combinations of conditions (such as depression and cardiovascular disease) [22], focus on a single approach for groups with MLTCs (e.g., mindfulness-based interventions) [23], expand their focus to all complex interventions [24], only consider physical LTCs in their scope [25], or only review one type of research design (e.g., a qualitative synthesis) [10]. To the best of our knowledge, no work to date has followed the increasingly endorsed AMS/NIHR definition of MLTCs, mapped out the evidence landscape across psychological approaches, nor charted its evolution over time.

To advance the field and guide future work, the primary objective of this scoping review is to systematically map the available evidence on psychological interventions for mental health of people living with MLTCs. By synthesising the existing research, we aim to provide a comprehensive overview of the types of interventions studied, the psychological outcomes targeted and the health conditions included in these studies. We also aim to chart the evolution of the research since the turn of the century, according to the Medical Research Council’s Phases of Complex Interventions Research: Intervention development/identification, Feasibility research, Effectiveness and Implementation [26]. Based on the current state of the literature, we offer recommendations for future research, clinical practice and policy on care of this growing population.

## Method

The research team developed a protocol following Arksey and O’Malley’s scoping review methodology [27] and pre-registered it (https://osf.io/qj947/). The review is reported in accordance with the PRISMA-ScR checklist (see S3 File) [28]. The funder of the study had no role in study design, data collection, data analysis, data interpretation or writing of the report.

We searched six databases (PubMed, Embase, Scopus, PsycNET, Web of Science, Cochrane; the latter also includes ClinicalTrials.gov and ICTRP) for records including keywords related to psychological interventions, long-term conditions and multimorbidity (see Table 1 for the search strategy). A search of reference lists, grey literature and hand search complemented the initial pool, although no eligible records were identified this way. To keep the review focused on quality-assessed work and capture the recent or current study of MLTCs, eligible studies had to be peer-reviewed papers published between January 2000 and January 2025 in English. After removal of duplicates, the first 10% of all titles and abstracts were independently screened by the first and senior authors against pre-specified eligibility criteria (see Table 2). In brief, eligible records reported on psychological interventions (defined as ≥50% of intervention time if the content was multi-modal) for adult samples with at least two LTCs (where a formal diagnosis of mental health conditions was not needed but a diagnostic cut-off on a validated measure was) and measured at least one validated mental health outcome. We included empirical research with primary or secondary data but excluded conference abstracts, trial registrations, unreviewed theses and commentaries. Review articles were deemed important to include as signals of interest and/or higher quantity in a particular area of MLTC interventions. Similarly, peer-reviewed protocols were included as an indicator of research to come—both of which aided our mapping of the literature’s evolution. The inter-rater reliability rate was excellent (96%) and conflicts were resolved in consultation with the wider research team. The first author then screened the remaining abstracts for consideration at full-text stage, where all publications were double-screened by the first and senior authors (85% agreement) and conflicts again resolved through discussion.

**Table 1:** Review search strategy.

Cluster	Search terms
1. Psychological interventions	(psych* AND interven*) OR cognitive behavio?ral OR cbt OR (behavioural AND activation) OR (behavioral AND activation) OR mindful* OR (dialectic* AND therap*) OR (talking AND therap*) OR counsel* OR psychodynamic OR (interpersonal AND therap*)
AND	
2. Multiple long-term health conditions	((long* AND term* AND condition*) OR (persist* AND health AND (issue* OR problem* OR condition*)) OR (chronic AND (disease OR condition)) AND ((comorbid* OR co-morbid* OR multimorbid*) OR (multiple OR “two or more” OR “2 or more”))
AND	
3. Research design	trial OR study OR controlled OR random* OR compar* OR pragmatic OR qualitative* OR implement* OR evaluat*

*Note.* Search conducted on records’ titles and abstracts, on records published between 01/01/2000 and 01/01/2025, in English language, for research on human adult participants. Exact search strategy syntax per database is given in S1 File.

**Table 2:** Inclusion and exclusion criteria.

	Include	Exclude
Participants	Adults (mean age 18 + years)	Infant, child, teen samples (aged <18)
	With two or more long-term/chronic health conditions (mental or physical, as per NIHR definition)	Samples with one long-term/chronic health condition where symptoms of another are only used as a measure (e.g., depression symptoms in sample with fibromyalgia; general statement of higher prevalence of distress in diabetes patients)
	Elevated symptoms or diagnostic cutoff is sufficient for mental health conditions (i.e., formal diagnosis not strictly necessary)	
Intervention	Psychological interventions, including cognitive-behavioural therapy, behavioural activation, interpersonal therapy, dynamic therapy and third-wave interventions (e.g., mindfulness)	Entirely behavioural interventions (e.g., physical activity programs, nutritional counselling)
	Delivered in any format, setting, medium, facilitation	Multi-modal interventions where the psychological component makes up <50% of intervention time
Comparator	Any or no control (no limits)	
Outcome	Any psychological health outcome measured on a validated psychometric questionnaire or another validated way (e.g., qualitative excerpts, EMA data)	
Study Design	Peer-reviewed studies reporting on interventions of most designs, including primary studies (quantitative, qualitative, mixed-methods; feasibility, pilot, full-scale intervention trials; case studies and case reports)	Non-peer-reviewed publications or other publication formats (e.g., PhD or MSc theses, commentaries and letters to editor, conference abstracts, trial registrations)
	Protocols of primary studies meeting all other criteria	
	Review articles, including systematic, rapid, scoping and narrative reviews meeting all other criteria	

Data extraction recorded the general study information (e.g., year of publication, country of first author), study design, MRC Framework stage [26] (*1. Intervention development/identification, 2. Feasibility/acceptability/pilot testing, 3. Effectiveness evaluation, 4. Implementation/economic evaluation*; if a study satisfied the criteria for multiple stages, such as a hybrid effectiveness-implementation design, it was counted towards all categories), participant conditions and how they were determined (*Self-report, Diagnostic assessment by clinician, Other*), number of participants, type of intervention as described by the authors (e.g., *CBT, ACT, Mindfulness, DBT, BA, Psycho-educational, Integrated/stepped care, Other*), the setting (*Community, Educational, Primary care, Healthcare other than primary care, Other*), delivery mode (*In person, Digital/remote, Mixed*), facilitator (*Primary care practitioner, Other healthcare practitioner (e.g., PwP), Dedicated study staff, Carer, Self-delivered, Other*), duration, frequency and descriptions of intervention content and any control conditions (as reported by authors). In terms of outcomes, we extracted the outcome domain (*Depression, Anxiety, Wellbeing, Stress, Self-rated health, Condition-specific impact on mental health (e.g., diabetes distress), Other*), validated measures used, how they were assessed (*Self-report questionnaire, Assessment by clinician, Other medical procedure*) and at what timepoints, and which groups were assessed (*Patients, Carers/family members, Healthcare providers, Policymakers, Other*). We recorded the direction of effect on mental health (*Improved, Mixed, No change, Deteriorated*), a summary of qualitative findings (as applicable), study limitations, funding sources and conflicts of interest, and any other notes. The extraction template was prepared in advance and piloted in the research team, which resulted in addition of fields recording the National Health Service Quality and Outcomes Framework condition groups (NHS QOF; i.e., categorising as *Cardiovascular, Respiratory, Musculoskeletal, Mental health and Neurology, Other LTCs (e.g., cancer, kidney disease)*) [29] on top of author-listed LTCs, and recommendations the extracted study makes for future work (categorised as *None specified, Cease research, Further research at this stage, Next stage research, Policy/practice change, Other*). Data extraction was completed by the first author, who has extensive experience with literature synthesis research, in consultation with the senior author where data were ambiguous. The full search strategy, extraction template and data are available in S1 and S2 Files, respectively.

Data synthesis involved organising the data in line with our review questions: 1) when, where and by whom the research was done, 2) which conditions did they include as LTCs, 3) which types of psychological interventions did they study, 4) which mental health outcomes were targeted and 5) what recommendations did the articles include for research, practice, and policy. Our interpretation was an iterative process led by the first author with input from the whole research team, guided by a summary spreadsheet with sections mapped on the review questions and colour-coded by MRC Stage of Evidence, research design, and geographical location. The synthesis and interpretation presented in the following sections are our attempt to answer the review aims (i.e., what evidence exists on psychological interventions for adults with MLTCs, how it is evolving over time and what it presently can(not) tell us). A narrative synthesis was deemed the most suitable to convey our results [30]. In line with best practice guidelines for scoping review methodology [27], we did not undertake formal statistical synthesis nor assess risk of bias, although only considering peer-reviewed publications from indexed journals served as a minimal quality threshold. Because review articles and primary studies were both eligible, we cross-checked the primary studies synthesised within each included review against those identified independently through our database searches. Five primary studies [31–35] appeared in both strands; they were retained and reported in both the primary and review-level syntheses without adjustment, since the two strands are presented separately and serve different mapping purposes.

## Results

### Study characteristics

The search and screening flowchart is given in Fig 1. Our final selection contains 54 studies reported in 60 publications and conducted across 15 countries (see Fig 2 for split by continent), with most lead authors based in the United Kingdom (n = 10), Australia (n = 10), United States of America (n = 9) and Spain (n = 7). Research designs included systematic reviews and meta-analyses of randomised controlled trials (RCTs; n = 7), primary RCTs (n = 16 reporting results and 11 protocols), pilot and/or feasibility studies (n = 14), retrospective observational studies (n = 3), case studies (n = 2) and economic evaluations (n = 2; one of which was a hybrid effectiveness-economic evaluation RCT). Context varied greatly between studies, however, there were clusters of research investigating remotely delivered interventions (e.g., telephone, videoconferencing, or mobile; n = 18), with older adult samples (n = 9) and from integrated care settings (i.e., programmes embedded within existing healthcare, such as those delivered by nurses or pharmacists; n = 18). Included research is summarised in Table 3, with more information in S2 File. Below, we describe the current primary evidence separately from review-level findings, which is summarised afterwards.

**Fig 1:**
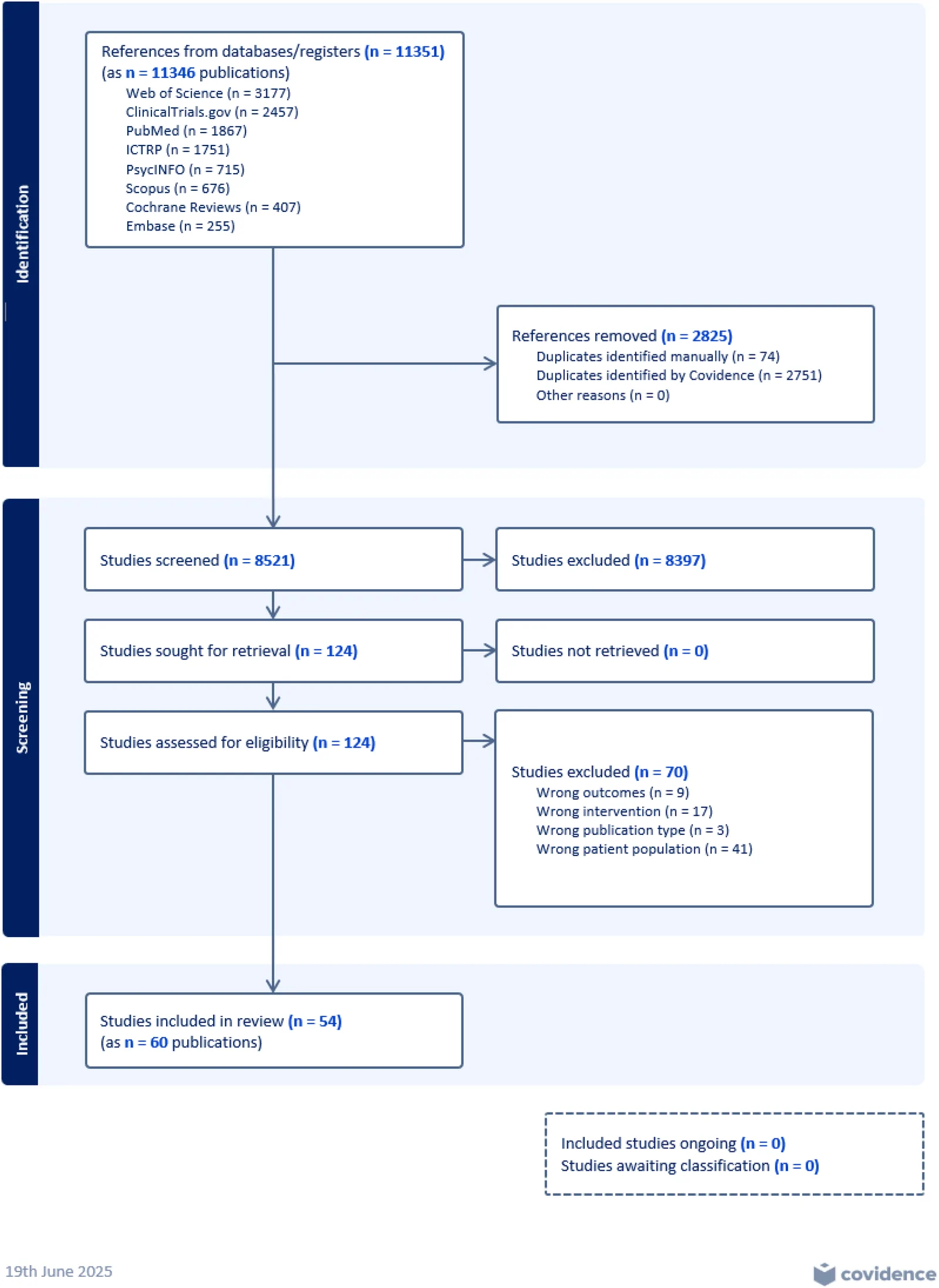
Preferred reporting items for systematic reviews and meta-analyses (PRISMA) flowchart.

**Fig 2:**
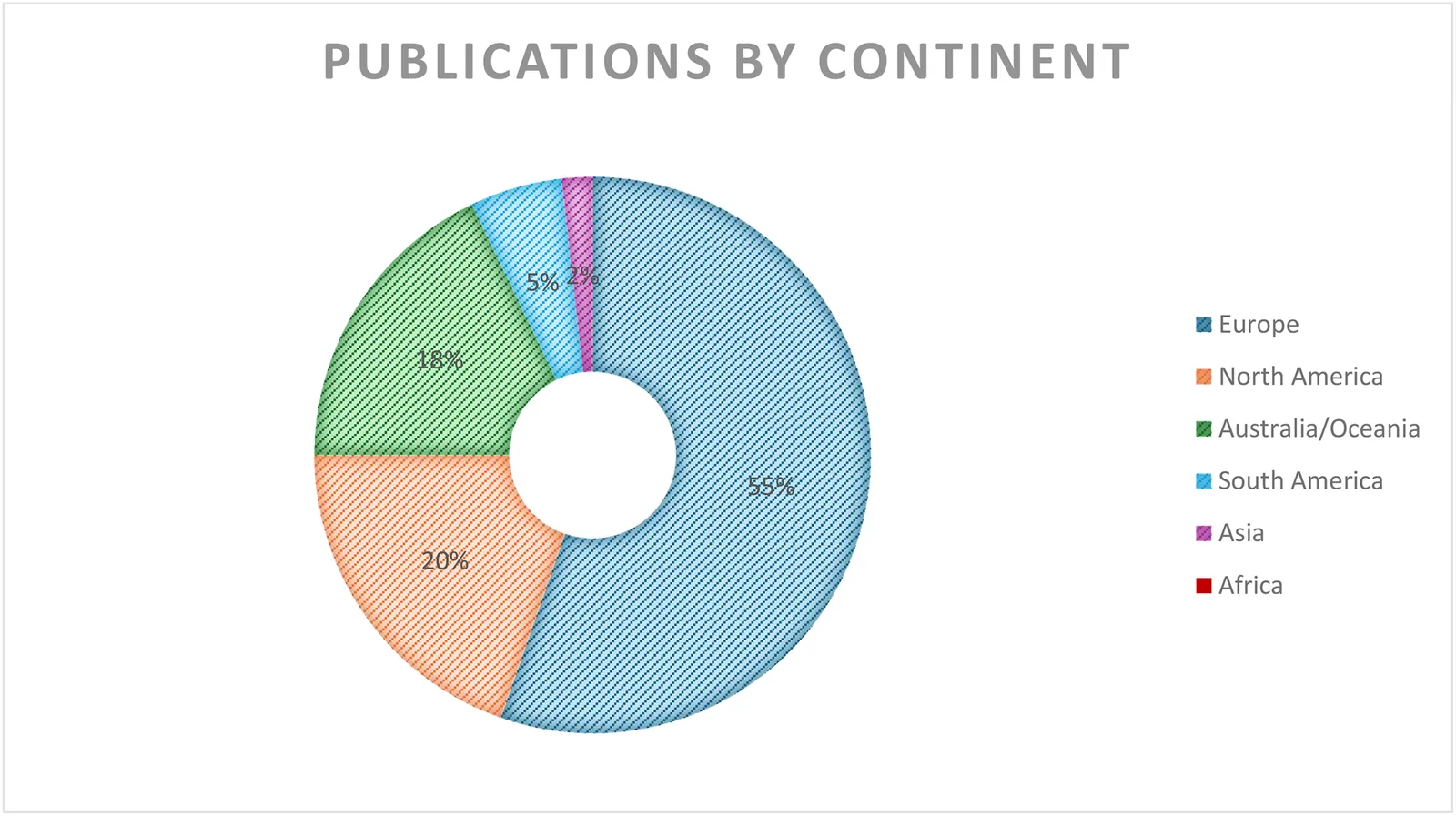
Geographic location of included studies by continent. *Note:* Location determined by the country of the first author’s institution. In the case of multiple institutions, the primary institution was used. There are currently no publications with primary author publications based in Africa, therefore the category has no corresponding section on the chart.

**Table 3:** Summary of the current research on psychological interventions for mental health in adults with MLTCs, broken down by study design.

Study type	Number	Countries	Conditions	Intervention	Outcomes	Context
Systematic review	7	Australia (n = 3)	Depression & any physical LTC (n = 1)	Range of psychological interventions (n = 4)	Depression & anxiety (n = 4)	High-income countries (n = 1)
		Canada (n = 1)	Depression/anxiety & COPD (n = 1)	CBT (n = 2)	Range of mental health outcomes (n = 2)	Older adults (n = 1)
		UK (n = 1)	Depression/anxiety & cardiovascular disease (n = 1)	CBT & MBI (n = 1)	Depression (n = 1)	Remote delivery (n = 2)
		USA (n = 1)	Depression & anxiety (n = 1)			
		Spain (n = 1)	Depression & diabetes (n = 1)			
			Depression & musculoskeletal pain (n = 1)			
			Depression/anxiety & chronic pain (n = 1)			
Economic evaluation	2	Australia (n = 1)	Depression & musculoskeletal pain (n = 1)	ACT & BA (n = 1)	Depression, anxiety, stress (n = 1)	Remote group delivery (n = 1)
		Spain (n = 1)	Depression/anxiety & COPD (n = 1)	CBT (n = 1)	Depression, anxiety, condition-specific impact (n = 1)	Remote individual delivery (n = 1)
RCT (completed)	16	Spain (n = 3)	Depression & any physical LTC (n = 5)	CBT (n = 5)	Depression, anxiety, condition-specific impact (n = 5)	Integrated care setting (n = 5)
		Australia (n = 2)	Depression & musculoskeletal pain (n = 3)	BA & psychoeducation (n = 4)	Depression, anxiety, quality of life (n = 4)	Older adults (n = 5)
		The Netherlands (n = 2)	Depression & diabetes (n = 2)	CBT, BA, psychoeducation (n = 3)	Range of mental health outcomes (n = 3)	Remote delivery (n = 3)
		UK (n = 2)	Depression/anxiety & COPD (n = 2)	MBI (n = 1)	Depression & self-rated health (n = 2)	COVID-19 measures (n = 1)
		USA (n = 2)	Depression & multiple sclerosis (n = 1)	ACT & BA (n = 1)	Depression & anxiety (n = 1)	Group delivery (n = 1)
		Germany (n = 1)	Depression/anxiety & respiratory disease (n = 1)	CBT & MBI (n = 1)	Depression & condition-specific impact (n = 1)	Pulmonary rehabilitation setting (n = 1)
		Italy (n = 1)	Depression/anxiety & gastrointestinal disease (n = 1)	CBT & DBT (n = 1)		
		Norway (n = 1)	Depression & diabetes/cardiovascular disease (n = 1)			
		Pakistan (n = 1)				
		Sweden (n = 1)				
RCT (ongoing)	11	Australia (n = 2)	Depression & any physical LTC (n = 4)	CBT, BA, psychoeducation (n = 3)	Depression, anxiety, condition-specific impact (n = 3)	Integrated care setting (n = 7)
		Spain (n = 2)	Depression & multiple sclerosis (n = 1)	CBT, BA, MBI, psychoeducation (n = 2)	Depression, anxiety, self-rated health (n = 2)	Remote delivery (n = 4)
		Brazil (n = 1)	Anxiety & personality disorders (n = 1)	CBT (n = 2)	Depression & anxiety (n = 2)	Group delivery (n = 2)
		Chile (n = 1)	Depression/anxiety & COPD (n = 1)	MBI (n = 1)	Depression & self-rated health (n = 2)	Planned economic evaluation (n = 2)
		Germany (n = 1)	Chronic pain & any other LTC (n = 1)	ACT (n = 1)	Depression & condition-specific impact (n = 1)	Older adults (n = 1)
		Ireland (n = 1)	Depression, chronic pain, HIV (n = 1)	CBT, BA, MBI (n = 1)	Range of mental health outcomes (n = 1)	
		Poland (n = 1)	Depression & diabetes/back pain (n = 1)	BA & psychoeducation (n = 1)		
		UK (n = 1)	Depression & diabetes/cardiovascular disease (n = 1)	Psychodynamic therapy (n = 1)		
		USA (n = 1)				
Pilot & feasibility (quantiative)	8	USA (n = 3)	Depression & any physical LTC (n = 3)	CBT, MBI, psychoeducation (n = 3)	Depression, anxiety, self-rated health (n = 2)	Remote delivery (n = 3)
		UK (n = 2)	Depression/anxiety & chronic pain (n = 2)	BA & psychoeducation (n = 3)	Depression & condition-specific impact (n = 1)	Older adults (n = 2)
		Canada (n = 1)	Depression & musculoskeletal pain (n = 1)	MBI & psychoeducation (n = 1)	Depression, anxiety, condition-specific impact (n = 1)	COVID-19 measures (n = 1)
		Germany (n = 1)	Depression & hypertension (n = 1)	MBI (n = 1)	Depression & anxiety (n = 1)	Integrated care setting (n = 1)
		The Netherlands (n = 1)	Depression & rhumatoid arthritis (n = 1)	CBT & MBI (n = 1)	Depression, anxiety, wellbeing (n = 1)	
				CBT, BA, psychoeducation (n = 1)	Depression, anxiety, quality of life (n = 1)	
					Depression (n = 1)	
Pilot & feasibility (qualitative & mixed-methods)	6	UK (n = 4)	Depression & diabetes/hypertension (n = 1)	ACT (n = 2)	Patient and provider experience (n = 5)	Remote delivery (n = 3)
		Australia (n = 1)	Depression & any physical LTC (n = 1)	CBT & BA (n = 1)	Acceptability (n = 2)	Integrated care setting (n = 3)
		Peru (n = 1)	Depression/anxiety & chronic pain (n = 1)	BA & psychoeducation (n = 1)	Preliminary efficacy (n = 2)	Healthcare provider perspective (n = 3)
			Depression & diabetes/cardiovascular disease (n = 1)	CBT, BA, psychoeducation (n = 1)	Resource-poor setting (n = 1)	
			Depression & multiple sclerosis (n = 1)	CBT (n = 1)		
			Depression/anxiety & gastrointestinal disease (n = 1)			
Retrospective observational study	3	Ireland (n = 1)	Personality disorder & eating disorder (n = 1)	CBT & DBT (n = 1)	Depression, affect, clinical diagnosis (n = 1)	Integrated care setting (n = 2)
		Spain (n = 1)	Depression & any physical LTC (n = 2)	CBT & psychoeducation (n = 1)	Depression & anxiety (n = 2)	Routine data use (n = 2)
		USA (n = 1)		CBT & BA (n = 1)	Remote delivery (n = 1)	
Case study	2	Australia (n = 1)	Depression & musculoskeletal pain (n = 1)	CBT, DBT, BA, ACT (n = 1)	Depression & condition-specific impact (n = 1)	
		Sweden (n = 1)	Panic disorder & COPD (n = 1)	CBT, MBI, BA, psychoeducation (n = 1)	Depression, anxiety, condition-specific impact (n = 1)	

### Conditions included as LTCs

In research reporting primary data, two main strands of evidence were identified. In the first, studies considered populations with a specific physical LTC—most commonly diabetes, [36,37] (COPD), [31,38,39] or chronic pain [32,33,35,40]—alongside a co-occurring mental health condition, typically depression. The interventions aimed to reduce psychological symptoms within the context of the primary physical illness rather than address the broader complexity of multimorbidity. Research in this strand often prioritised feasibility and effectiveness in narrowly defined populations.

In the second strand, studies adopted a more inclusive definition of MLTCs, involving participants with two or more chronic physical and/or mental health conditions, regardless of the specific combination. These interventions recognised the cumulative and interactive effects of multiple conditions. For instance, Gilbody and colleagues [41] included participants with two or more physical LTCs and at least mild depression, while Chew-Graham and colleagues [42] targeted individuals with a broad range of chronic somatic diseases and depression. Some studies explicitly designed interventions for any LTC with co-morbid depression [43–45], while others allowed inclusion of participants with any two or more LTCs [46], reflecting a shift towards approaches that address multimorbidity as a condition unto itself. This more inclusive definition was common in integrated care settings and enabled the exploration of transdiagnostic methods and broader generalisability.

Depression was the most studied condition, required in all but three studies [47–49] (94%). Anxiety (24%), stress and psychological distress (6%) were less frequently studied as contributing conditions. Other LTCs include multiple sclerosis [50–52], inflammatory bowel disease [53,54], human immunodeficiency virus [55] and rheumatoid arthritis [56], all still alongside depression. A minority of studies defined eligible LTCs as physical conditions only [49], highlighting differing conceptualisations within a field of research that are not always apparent from the terminology used. One study did not report their definition of LTCs [57].

### Types of interventions

Studies evaluated a range of psychological intervention approaches. The most frequent approaches were CBT (62% of studies) and BA (70%), either as a standalone intervention (CBT in 4% [51,52] and BA in 4% [41,42]) or a core component in multi-modal programmes. They were often combined with each other or with psychoeducation, particularly in interventions targeting depression comorbid with diabetes, [37] cardiovascular disease [58] or COPD [31,39].

MBIs also appeared frequently (30%), although combined with other modalities in all but one study [59]. They were present either as structured mindfulness-based cognitive therapy [45,59], mindfulness-based stress reduction [56] or through third-wave approaches such as acceptance and commitment therapy (ACT; 13%) [35,48,60]. The latter were particularly common in trials involving chronic pain [48,60] or mixed LTC populations [57].

Most current interventions (85%) consist of multiple approaches, blending various evidence-based strategies—such as CBT, mindfulness, BA and psychoeducation—into a single programme [36,40,43,61–65]. These were frequently tailored to complex patient presentations, including those with several LTCs and overlapping psychological distress. While prevalent and promising, these interventions varied considerably in content and structure, making cross-study comparisons challenging.

Intervention duration ranged from four to 24 weeks, with some offering an initial high-intensity period followed by a less involved maintenance phase [40,58]. The most common session frequency was weekly, although several studies offered flexible schedules. An outlier in frequency was Suszek and colleagues’ trial [66] with daily sessions in an in-patient setting. Modes of delivery were split between in-person (51%), remote (i.e., telephone, video-conferencing, or digital platforms; 26%) and mixed delivery (21%). Interventions were delivered by dedicated study staff (26%) [31,34,41,50], primary care providers or nurses (19%) [43,61,67,68], other healthcare professionals (e.g., psychologists or PwPs; 62%) [33,36,38,44,53] or self-delivered (9%) [46,48,51,69] alone or in combination—in total, just under a quarter involved multiple delivery providers [35,62,67,70]. A handful of studies reported using innovative facilitators, such as community pharmacists [42], osteopaths [60] or mindfulness teachers [57]. Both individual and group-based formats were reported, with group sessions more common in remotely delivered or resource-strained systems, such as primary care [34,35,61].

A subset of studies evaluated integrated care or stepped care models (33%), where psychological support was embedded within broader healthcare provision [33,44,55,62,64,71]. These approaches were typically used in primary care or chronic disease management settings, often targeting populations with diabetes, cardiovascular disease or COPD—which account for a significant proportion of all primary care consultations [4]. Interventions in these contexts emphasised accessibility, continuity of care and tailoring to individual patient needs, usually starting with low-intensity support and escalating as required.

### Mental health and wellbeing outcomes

Included studies prioritised mental health symptoms, particularly depression and/or anxiety, as primary outcomes, though several also assessed outcomes like stress, quality of life and condition-specific impacts. Outcomes were predominantly measured through self-report questionnaires, with a few studies incorporating clinician-administered assessments [31,36,50,63].

Depression was the most commonly assessed outcome (89% of studies), consistent with its high prevalence among individuals with MLTCs [6]. Studies employed a range of validated self-report tools, with the Patient Health Questionnaire-9 (PHQ-9) being the most frequently used [37,38,41,42,59,67,68,72], followed by the Beck Depression Inventory (BDI-II) [31,45,47,50,61,63] and the Hospital Anxiety and Depression Scale (HADS-D) subscale [44,53]. In a smaller number of studies, clinician-rated tools such as the Hamilton Depression Rating Scale (HAM-D) were used [36,67]. Depression was the primary outcome of interest in interventions targeting diabetes [36], cardiovascular disease [68] or chronic pain [34,35], and was used to assess both symptom severity and treatment response over time.

Anxiety was the second most common outcome (69%). It was measured via the Generalized Anxiety Disorder 7-item scale (GAD-7) [34,36,41,42,45,49,68], the HADS-A subscale [32,53,59], Beck Anxiety Inventory (BAI) [31,38,50] or the State-Trait Anxiety Inventory (STAI) [52,66]. It frequently appeared as a secondary outcome in studies where interventions were not explicitly designed to target anxiety.

A handful of studies included measures of stress or psychological distress (7%), using tools such as the Perceived Stress Scale (PSS) [50] or as part of a holistic mental health assessment in the Depression, Anxiety and Stress Scales (DASS-42 or DASS-21) [35,54]. These appeared in multi-component interventions addressing emotional wellbeing or where assessing impact on caregivers or healthcare providers [37,50].

Measures of quality of life—both mental and physical—were also included (35%), often using the Short Form Health Survey (SF-12 or SF-36) [43,44,53,67] or EuroQoL-5D (EQ-5D) [41,54,61]. Other studies assessed mental wellbeing (6%), with scales including the World Health Organization-5 Wellbeing Index [36,45] or the Warwick-Edinburgh Mental Wellbeing Scale (WEMWBS) [39]. These outcomes provided a more holistic perspective on intervention impact but were typically secondary to symptom-based measures.

Finally, several studies assessed condition-specific psychological impacts, such as disease-specific quality of life (11%) [38,56,68], diabetes distress (4%) [36,37] or pain-related functional interference (17%) [33–35,73].

Non-mental health-related outcomes assessed healthcare and social participation (treatment adherence [37,55], healthcare utilisation [43,64], time off work [32], inflammation biomarkers [56], glycaemic control [36]), behavioural measures, (physical activity [37,55] or sleep [31,52]) or hypothesised mechanisms driving mental health improvement (mindfulness [45,57], emotion regulation [47,63], self-efficacy [54], self-compassion [45], resilience [54], loneliness [41,49]).

### Review-level findings

Seven systematic reviews met the inclusion criteria, all of which mapped onto Stage 3 (Effectiveness evaluation) of the MRC framework. These reviews mostly synthesised data across populations defined by a single physical long-term condition paired with a comorbid mental health issue. Specifically, the reviews focused on depression and/or anxiety co-occurring with chronic obstructive pulmonary disease (COPD) [74]; chronic musculoskeletal pain [73]; generic chronic pain [75] and diabetes [76]. Three reviews broader in scope evaluated dyads of chronic somatic conditions and anxiety/depression [77,78] or focused purely on psychiatric co-occurring mood and anxiety disorders in older adults [79]. The reviewed intervention modalities ranged from any psychological interventions in four reviews [74,76,77,79] to a narrower scope of CBT-based interventions [75], remotely delivered interventions [73] or remotely-delivered CBT [78]. While depression and anxiety were universally tracked as the primary outcomes, several reviews also captured impacts on stress, wellbeing, self-rated health and condition-specific functional impacts [77,78].

In terms of future work, review-level evidence recommended shifts toward policy and clinical practice changes to integrate these interventions into routine care pipelines [73,74,77]. Concurrently, authors underscored the need for primary evidence at the fourth MRC Stage (i.e., Implementation research) [73,77], alongside more rigorous effectiveness trials within their respective populations [75,76,79]. Finally, review-level literature highlighted the necessity of evaluating diverse, socioeconomically varied cohorts within low- and middle-income countries [78].

## Discussion

### Summary of findings

This scoping review mapped the current evidence on psychological interventions for mental health in adults living with MLTCs. We identified 54 studies spanning a broad range of intervention types, study designs and target populations. Most interventions focused on managing co-morbid depression in the context of a specific physical condition, with CBT, BA and mindfulness-based approaches being the most frequently evaluated. While the number of feasibility and effectiveness trials based in Europe and USA has expanded over the past decade, only a small number of studies incorporated economic evaluation or focussed on implementation. The most frequent mental health outcomes were depression and anxiety, with a growing interest in broader wellbeing, condition-specific impacts and mechanisms of change. Despite progress, the literature remains underdeveloped in key areas of complex presentations, inclusivity and real-world application.

### Evolution of evidence base

Research on psychological interventions for mental health in adults with MLTCs has steadily increased over the past two decades. The earliest eligible studies emerged in the mid-2000s but momentum began building after 2015. This acceleration likely reflects the growing recognition of multimorbidity as a public health priority [21] and associated increased investment in complex intervention research [19,21].

When mapped against the MRC framework for complex interventions [26] (see Fig 3), the majority of studies fall into Stage 3 (Effectiveness), indicating that some of the field has moved beyond early-stage development. Specifically, over one-third of included studies were RCTs. Yet, new interventions are still being developed (and are rarely published as Stage 1 papers), so feasibility trials remain common. While the growth in Stage 3 evidence is encouraging, it is mostly focused on efficacy in controlled settings instead of effectiveness and economic evaluations. Only two published studies to date evaluated cost-effectiveness [80,81] and a handful of protocol papers indicate that health economic assessments are forthcoming [39,52,65]. Evidence of this type is crucial for policymakers and advocates when demonstrating the value of MLTC interventions at scale, so its lack is likely a limiting factor in the translational pathway. At present, Stage 4 (Implementation and Dissemination) evidence is lacking. None of the included studies focused on implementation or dissemination, revealing another gap between evidence of intervention efficacy and real-world integration.

**Fig 3:**
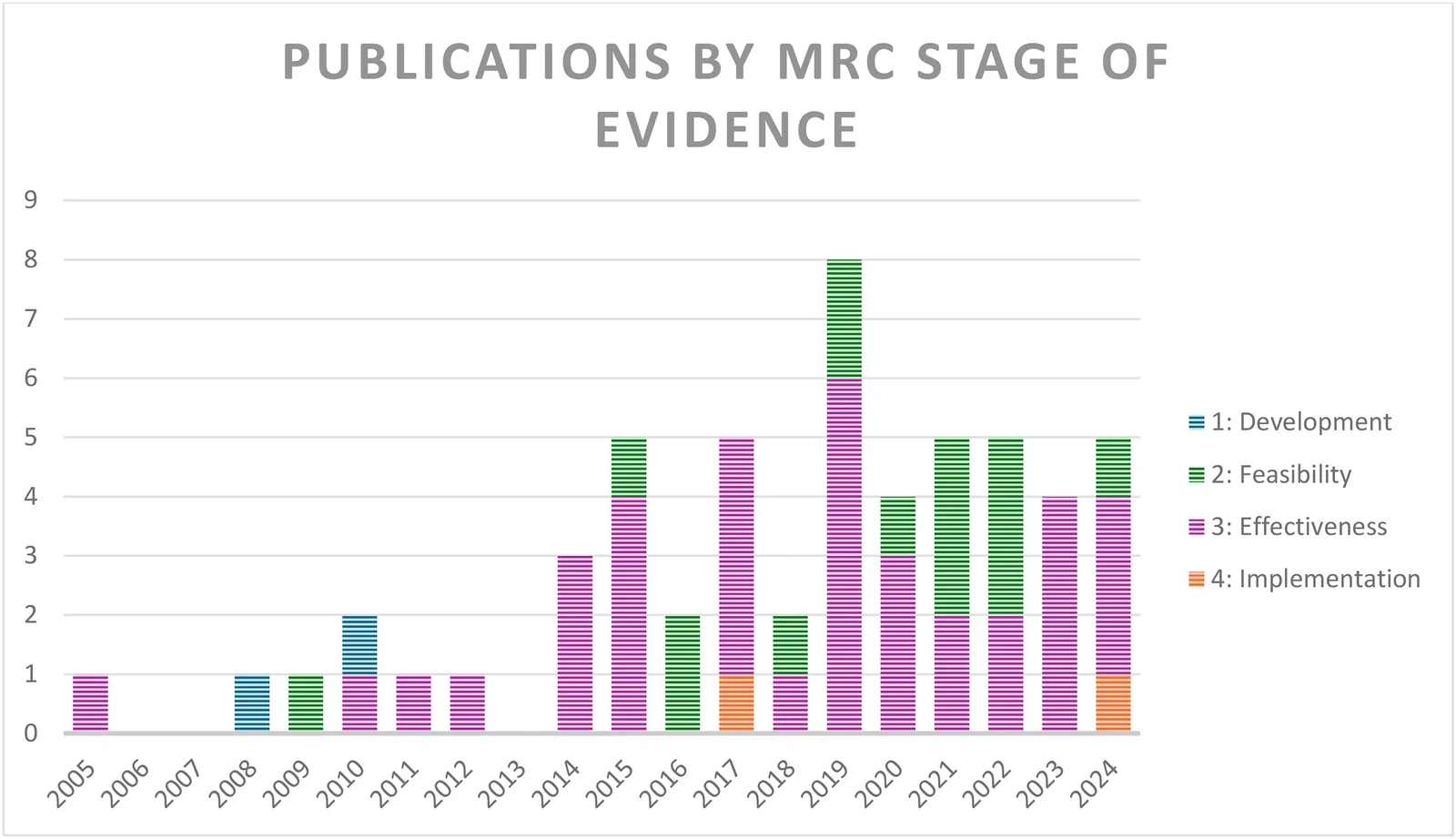
Evolution of the evidence base by Medical Research Council’s Complex Interventions Framework (Skivington et al., 2021) over time.

Notably, the progression of research through MRC stages is not evenly distributed. Interventions embedded within integrated or stepped-care models, often conducted in high-income settings, appear more likely to progress toward full-scale trials and economic evaluation. However, studies addressing service-level barriers, workforce capacity or equity of access are lacking in this context too.

### Comparison with previous literature

Our findings largely align with existing literature, which emphasises that the burden of MLTCs is only beginning to be understood and that successful interventions for it are likely to be “complex and multifaceted” [3]. The problem of an inconsistent definition for multimorbidity (and a call for reporting requirements) has been empirically demonstrated in a recent review [82]. The overwhelming focus on co-morbid mental health conditions in MLTC research may be explained by the crucial role these conditions play both in increasing one’s risk factor for developing MLTCs [6,7] and in worsening the prognosis of people with MLTCs [83]. Previous work has also identified non-random clustering patterns of conditions under the broader umbrella of MLTCs, which aligns with the foci of interventions we reviewed (i.e., key clusters being cardiovascular, mental health-related and musculoskeletal) [84]. This data-driven approach to patterns of disease co-occurrence is the first step towards identifying common, mechanistically related and prognostically relevant clusters that are amenable to psychological intervention, and thus deserves attention in future work [85].

Nevertheless, this review both builds on and challenges existing research. First, it highlights the increasing importance of integrated healthcare, with multidisciplinary teams and shared-care approaches. Our mapping captured the promise of intervention delivery within unexpected settings and by non-traditional providers—such as community pharmacists [42] and osteopaths [60]—illustrating a shift toward pragmatic, embedded physical-mental healthcare models that prior reviews have largely overlooked. Secondly, our broad criteria for eligible designs and intervention types highlights the varied array of approaches being considered for mental health management in groups with MLTCs. Given the burden and growing prevalence of this public health issue, collating all these attempts in one place—from published case studies to forthcoming economic evaluations—allows a helpful overview of the topic. Lastly, while earlier reviews primarily focused on establishing clinical efficacy of interventions, mapping the wider evidence base against the MRC framework allows us to highlight gaps across it, including the reasons for their existence. Our review demonstrates that the field is not stalling due to a lack of primary effectiveness data (Stage 3), but rather due to a young and developing evidence base focused specifically on multimorbidity as a condition unto itself, paired with absence of structural support for implementation and dissemination research (Stage 4) and health economic evaluations.

### Recommendations for research, policy and practice

The developing evidence base in the area of MLTCs management is encouraging, although much work remains to be done. Currently, most studies focus on effectiveness (MRC Stage 3) and a growing number progress beyond feasibility or pilot phases. However, there is a clear skew towards addressing fixed combinations of conditions and assessing impact only on patients. Future studies should incorporate the perspectives of healthcare practitioners, caregivers and family members. Further, much of the published literature remains exploratory rather than practice-changing. We present the key gaps in literature below and summarise our recommendations for research, policy and practice in Table 4.

**Table 4:** Recommendations and future directions.

Research	
	Standardise definitions and outcome measures for cross-study comparability.
	Report complex interventions thoroughly, ideally following a standardised classification framework (e.g., Behaviour Change Taxonomy [86] or Ontology [87]) to aid study replication and evidence synthesis.
	Conduct implementation and scale-up studies to assess real-world effectiveness and sustainability.
	Design person-centred interventions that address the full complexity of multimorbidity.
	Include underserved populations and settings, including low-income and global South contexts.
Policy	
	Integrate psychological care into routine MLTC pathways across health systems.
	Fund Stage 4 trials and hybrid implementation-effectiveness studies.
	Support interdisciplinary collaboration through workforce and training initiatives.
	Develop policies to ensure equitable access for marginalised MLTC populations.
Practice	
	Implement flexible, modular interventions adaptable to individual needs.
	Train healthcare staff to deliver low-intensity psychological support.
	Embed routine screening for psychological distress in MLTC care.
	Utilise digital tools to expand access to psychological interventions.

A major challenge identified in this review is the lack of consistency in how MLTCs are defined across studies. Some authors define MLTCs strictly as two or more physical conditions, while others include co-morbid mental health diagnoses such as depression or anxiety. Still others select their own assortment of LTCs of interest or refer to “chronic conditions” without specification. These inconsistencies make it difficult to compare findings or synthesise evidence across studies. To advance the field, future research should adopt a standardised definition—such as that from the UK Academy of Medical Sciences [21] and NIHR [19]—which includes physical non-communicable conditions, long-duration mental health conditions and long-term infectious diseases. A shared conceptual foundation will improve both comparability and translational relevance [82].

A key priority for future research is improving the transparency and consistency of intervention reporting. Greater use of established taxonomies and reporting frameworks (e.g., TIDieR [88], The Behaviour Change Ontology [87]) would enable clearer specification of intervention components, facilitate replication and strengthen the quality of evaluation and evidence synthesis. Enhanced reporting standards would also support more meaningful comparisons across studies and improve our ability to identify which components drive effectiveness in complex interventions.

In terms of intervention content, the field of MLTCs is extending evidence from single-LTC settings in support of established modalities, particularly CBT, BA and mindfulness-based interventions (e.g., MBCT). These approaches show consistent benefits in reducing depression, anxiety and psychological distress in people with co-existing LTCs [13–15,22]. In parallel, multi-modal programmes (often integrating CBT, psychoeducation, mindfulness and lifestyle components) are being developed and evaluated as alternatives, especially in primary care and digital settings. While these newer models are still being tested for feasibility and scalability, they likely represent the future direction of MLTC-focused psychological care due to their flexibility and fit within stepped or integrated care systems.

The context in which research is conducted also reveals important limitations. Nearly all included studies were conducted in high-income countries, particularly in Western Europe, North America and Australia. Samples were predominantly drawn from urban, majority-ethnic and higher socioeconomic status (SES) populations. This is in stark contrast to the global distribution and burden of MLTCs, which disproportionately affects people from lower SES backgrounds [4,89] and rising sharply in low- and middle-income countries [2,21]. A future priority must be the inclusion of more diverse and representative populations, such as rural samples and research in the Global South—both to enhance equity and to ensure generalisability of findings.

Finally, while the literature base is maturing in terms of intervention efficacy, it is still underdeveloped in implementation and dissemination research. Only a small number of studies have addressed MRC Stage 4 objectives and few have focused on long-term adoption or systems-level integration. This gap may reflect structural barriers, such as fragmented service delivery, limited funding or lack of implementation expertise in research teams [90,91]. Implementation science is essential not only for embedding effective interventions into healthcare systems but also for demonstrating value to policymakers and funders [17,19]. Future research should, where warranted by prior evidence, prioritise pragmatic trials, health economic evaluations and real-world effectiveness studies in diverse settings [92]. This shift will be key to advancing policy impact and improving outcomes for people living with MLTCs.

### Strengths and limitations

This review was pre-registered, conducted by a team including clinician-researchers and followed rigorous scoping review methodology and open science principles. A key limitation is the focus on studies that explicitly referenced MLTCs or multimorbidity, which may have excluded relevant research on single conditions with co-occurring mental health issues (e.g., psychological interventions for adults with cardiovascular disease and depression) [22]. This was a deliberate choice to assess literature treating MLTCs as a distinct clinical concept, but it narrows the scope of our conclusions. Similarly, the exclusion of lifestyle-focussed and self-management interventions, such as adapted exercise programmes [93,94], means the review focusses strictly on interventions with a primary psychological component. We mapped intervention approaches into broad categories (CBT, MBIs, BA, psychoeducation and integrated care) without following a structured classification system. Our approach to grouping the interventions was directly guided by, and limited by, the level of detail provided in the primary papers. To avoid over-interpretation or mischaracterisation, we chose to classify the interventions based on the terms and descriptions used by the primary authors themselves. A future direction—dependent on sufficiently detailed reporting—would be to map evidence and its hypothesised mechanisms of action according to granular intervention classification systems (e.g., the Behaviour Change Ontology [87]), which was presently not possible. Our title/abstract screening and data extraction were not done in full by two independent scorers; instead, the process was piloted on 10% of the papers at each stage, then one author completed the work and highlighted any ambiguous records to discuss with the full team. While this process was efficient and maintained the strength of multiple perspectives, it lacks the rigour of independent double screening and extraction. The lack of formal quality assessment, while in line with best-practice guidelines, means that our recommendations are based on all available evidence rather than prioritising the highest-quality outputs only. Relatedly, a small number of primary studies (five) were captured both independently and within the included reviews, three of which derived from a single recent review [75]. This overlap was modest and, given that primary and review-level evidence are reported as separate strands, it does not substantially change the interpretation of the evidence map. Finally, because the review focused on interventions targeting mental health outcomes, its recommendations may not apply to those aimed primarily at physical health or underlying psychological mechanisms—areas that warrant future complementary reviews.

## Conclusion

This work provides an overview of psychological interventions targeting mental health outcomes in individuals with MLTCs. For clinical practice, the findings support the use of evidence-based psychological interventions—particularly CBT, BA and mindfulness—for improving mental health in MLTC populations. However, the limited diversity of study samples and narrow focus on fixed condition combinations suggest a need for more inclusive, flexible and person-centred approaches in routine care. From a policy perspective, the review underscores the importance of investing in integrated care models that address both physical and mental health needs, and in implementation and cost-effectiveness research. In terms of future study, there is a clear need for adopting standardised definitions of MLTCs, including underrepresented populations and moving beyond efficacy to explore real-world impact. Prioritising pragmatic trials, economic evaluations and system-level integration will be essential to translating evidence into meaningful improvements in care and outcomes for people living with MLTCs.

## Supporting information

S1 File Scoping review search strategy per database. (DOCX)

S2 File Scoping review extracted data. Tab ‘Covidence extracted’ gives the raw data extracted from the review software Covidence. Tab ‘Summary’ reports initial grouping per study design and stage of evidence, which was used as the basis for team discussion and interpretation.
(XLSX)

S3 File Preferred Reporting Items for Systematic reviews and Meta-Analyses extension for Scoping Reviews (PRISMA-ScR) Checklist. (PDF)
