# Supplementary material for: Psychological interventions for mental health in adults with multiple long-term conditions: A scoping review and evolution of the evidence base
Source: PLoS One. 2026 Oct 13;21(10):e0355366. doi: 10.1371/journal.pone.0355366 (PMC13660903; doi:10.1371/journal.pone.0355366)
Supplement: Supplementary file 2 [file pone.0355366.s001.docx]

Thank you for the opportunity to review this paper. The manuscript presents a scoping review of psychological interventions for mental health in adults living with multiple long-term conditions (MLTCs). The topic is timely and relevant given the increasing global burden of multimorbidity and its associated mental health challenges. The review is generally well-written, methodologically informed, and draws on a substantial and diverse body of literature.

Below are my comments/suggestions the authors may find useful for improving the overall quality of the paper.

**Abstract**

The abstract methods section is quite scanty and could be strengthened by providing additional methodological detail. The authors may briefly describe how studies were selected and screened, including the study selection process and any eligibility procedures used. It may also be useful to indicate the methodological framework or reporting guideline adopted for the scoping review (e.g., PRISMA-ScR), as well as the types of studies included

**Introduction**

Although the introduction repeatedly states that the evidence base is “fragmented,” “underdeveloped,” or “underexplored,” the specific knowledge gap remains somewhat broad. The authors may more clearly specify what is currently unknown, why existing evidence syntheses are insufficient, and how this scoping review uniquely advances the literature. For instance, it is unclear whether previous reviews excluded MLTC populations, focused only on specific psychological interventions, or failed to examine the evolution of the evidence base over time.

**Methods**

1. Although the authors indicate that the review followed Arksey and O’Malley’s framework and PRISMA-ScR guidelines, the eligibility criteria are not sufficiently described within the methods narrative. The authors should clearly specify the inclusion and exclusion criteria beyond referring readers to Table 1, including eligible study designs, publication types, language restrictions, and whether grey literature was considered.

2. The search strategy section could be improved by indicating whether reference list screening, citation tracking, hand-searching, or grey literature searches were conducted. Given the evolving nature of MLTC research, omission of these sources may limit comprehensiveness.

3. The data charting and synthesis process is described only briefly and requires further clarification. The authors should provide more detail on how data extraction/charting was conducted, whether the extraction process was performed independently by more than one reviewer, whether the extraction form was piloted or refined iteratively during the review process, and how disagreements arising during data extraction were resolved

**Results**

The categorization of evidence strands requires further clarification. The distinction between studies focusing on a specific LTC plus depression and those adopting broader MLTC definitions is useful, but the criteria used to classify studies into these strands are not fully explained.

**Discussion**

The “Comparison with previous literature” section appears somewhat selective and primarily confirmatory, with limited critical engagement. Although it links the review findings to existing studies, it does not sufficiently interrogate, contrast, or explain inconsistencies within the broader literature. In particular, the section would be strengthened by a more explicit discussion of areas where the current findings diverge from prior reviews, as well as a deeper exploration of why certain anticipated evidence domains—such as more robust implementation research or greater diversity in intervention approaches—remain underdeveloped or absent.
