# Supplementary material for: Psychological interventions for mental health in adults with multiple long-term conditions: A scoping review and evolution of the evidence base
Source: PLoS One. 2026 Oct 13;21(10):e0355366. doi: 10.1371/journal.pone.0355366 (PMC13660903; doi:10.1371/journal.pone.0355366)
Supplement: Peer review history

# Peer review: Decision Letter 0

**DOI:** 10.1371/journal.pone.0355366.r001

**Authors:** Dorothy Serwaa Boakye

14 May 2026

Dear Dr. Lambert,

Thank you for submitting your manuscript to PLOS ONE. After careful consideration, we feel that it has merit but does not fully meet PLOS ONE’s publication criteria as it currently stands. Therefore, we invite you to submit a revised version of the manuscript that addresses the points raised during the review process.

Please submit your revised manuscript by Jun 28 2026 11:59PM. If you will need more time than this to complete your revisions, please reply to this message or contact the journal office at plosone@plos.org. When you're ready to submit your revision, log on to https://www.editorialmanager.com/pone/ and select the 'Submissions Needing Revision' folder to locate your manuscript file.

- A letter that responds to each point raised by the academic editor and reviewer(s). You should upload this letter as a separate file labeled 'Response to Reviewers'.
- A marked-up copy of your manuscript that highlights changes made to the original version. You should upload this as a separate file labeled 'Revised Manuscript with Track Changes'.
- An unmarked version of your revised paper without tracked changes. You should upload this as a separate file labeled 'Manuscript'.

If you would like to make changes to your financial disclosure, please include your updated statement in your cover letter. Guidelines for resubmitting your figure files are available below the reviewer comments at the end of this letter.

If applicable, we recommend that you deposit your laboratory protocols in protocols.io to enhance the reproducibility of your results. Protocols.io assigns your protocol its own identifier (DOI) so that it can be cited independently in the future. For instructions see: https://journals.plos.org/plosone/s/submission-guidelines#loc-laboratory-protocols. Additionally, PLOS ONE offers an option for publishing peer-reviewed Lab Protocol articles, which describe protocols hosted on protocols.io. Read more information on sharing protocols at https://plos.org/protocols?utm_medium=editorial-email&utm_source=authorletters&utm_campaign=protocols.

As the corresponding author, your ORCID iD is verified in the submission system and will appear in the published article. PLOS supports the use of ORCID, and we encourage all coauthors to register for an ORCID iD and use it as well. Please encourage your coauthors to verify their ORCID iD within the submission system before final acceptance, as unverified ORCID iDs will not appear in the published article. *Only*  the individual author can complete the verification step; PLOS staff *cannot*  verify ORCID iDs on behalf of authors.

We look forward to receiving your revised manuscript.

Kind regards,

Dorothy Serwaa Boakye, PGDE,MA,Mphil, RN, BSc

Academic Editor

PLOS One

Journal requirements:

When submitting your revision, we need you to address these additional requirements.

1. Please ensure that your manuscript meets PLOS ONE's style requirements, including those for file naming. The PLOS ONE style templates can be found at

https://journals.plos.org/plosone/s/file?id=wjVg/PLOSOne_formatting_sample_main_body.pdf and

https://journals.plos.org/plosone/s/file?id=ba62/PLOSOne_formatting_sample_title_authors_affiliations.pdf

2. Please include a separate caption for each figure in your manuscript.

3. Please include your tables as part of your main manuscript and remove the individual files. Please note that supplementary tables should remain as separate "supporting information" files.

4. Please include captions for your Supporting Information files at the end of your manuscript, and update any in-text citations to match accordingly. Please see our Supporting Information guidelines for more information: http://journals.plos.org/plosone/s/supporting-information.

If the reviewer comments include a recommendation to cite specific previously published works, please review and evaluate these publications to determine whether they are relevant and should be cited. There is no requirement to cite these works unless the editor has indicated otherwise.

[Note: HTML markup is below. Please do not edit.]

Reviewers' comments:

Reviewer's Responses to Questions

**Comments to the Author**

1. Is the manuscript technically sound, and do the data support the conclusions?

Reviewer #1: Yes

Reviewer #2: Yes

**********

2. Has the statistical analysis been performed appropriately and rigorously?

Reviewer #1: N/A

Reviewer #2: N/A

**********

3. Have the authors made all data underlying the findings in their manuscript fully available?

The PLOS Data policy

Reviewer #1: Yes

Reviewer #2: Yes

**********

4. Is the manuscript presented in an intelligible fashion and written in standard English?

Reviewer #1: Yes

Reviewer #2: Yes

**********

Reviewer #1: This manuscript addresses a timely and important public health topic. The use of the MRC Complex Interventions Framework to chart the evolution of the evidence base is a strength. However, several fundamental methodological concerns must be addressed before the manuscript can be considered for publication.

MAJOR CONCERNS

1. Inclusion of Systematic Reviews as Primary Data Sources

Seven systematic reviews and meta-analyses are included among the 54 studies and treated as equivalent to primary studies during data extraction. This is methodologically problematic for three reasons: it introduces a unit-of-analysis error by conflating aggregated and primary evidence; it risks double-counting primary studies that may have been captured both within those reviews and independently through the authors' own database searches; and it means findings are extracted from already-interpreted data, adding an additional layer of potential bias. The authors must either exclude systematic reviews from the evidence synthesis and use them only to inform the background and discussion, or provide explicit methodological justification for their inclusion with a clear description of how overlap with primary studies was managed. If reviews are retained as a distinct evidence category, this should be clearly labelled and handled as a separate strand of the synthesis.

2. Methods Section is Insufficiently Detailed

The Methods section is too brief for a scoping review. Several critical details are missing. The full search strategy is not presented in the manuscript body as required by PRISMA-ScR; relegating it entirely to supplementary files is insufficient. The rationale for the January 2000 start date is not explained. The data extraction process is not adequately described — it is unclear whether extraction was conducted by one or two reviewers, and no inter-rater reliability figure is provided for extraction (only for screening). The charting and synthesis process is described in one sentence and does not explain how data were coded or categorised against the five review questions. These omissions need to be addressed in full.

3. Discrepancy Between the Abstract, Methods, and PRISMA Flow Diagram

The abstract and Methods section state that six databases were searched, but the PRISMA flow diagram lists eight sources, including ClinicalTrials.gov and ICTRP. This inconsistency must be resolved. If trial registries were searched, this should be stated in the Methods and the implications for screening described. If they were not, the flow diagram requires correction.

4. Numerical Inconsistencies in Study Counts

The study design categories listed in the Results section sum to 55, not 54 as reported. This discrepancy must be identified and corrected across the manuscript, tables, and flow diagram.

5. Absence of Any Quality Consideration

While quality appraisal is not mandatory in scoping reviews, the authors draw direct clinical and policy recommendations from their findings. In this context, the complete absence of any quality consideration is difficult to justify. The authors should at minimum acknowledge this explicitly as a limitation and discuss how it affects confidence in their recommendations.

6. MRC Stage Classification is Not Explained

The assignment of studies to MRC stages is central to one of the paper's key contributions, yet no decision rules or criteria are described. Who made these assignments, on what basis, and were they checked independently? Without this, Figure 3 and the associated narrative cannot be evaluated.

CONCERNS ABOUT THE RESULTS SECTION

7. Results Read as a Narrative Review Rather Than a Scoping Review

The Results section presents findings through interpretive prose rather than structured mapping, which is more characteristic of a narrative review. Statements describing "emerging trends" and intervention emphases reflect the authors' synthesised reading of the literature and belong in the Discussion. The Results section should present a transparent, quantified account of what was found, reserving interpretation for later.

8. Interventions Are Not Classified Using a Recognised Framework

This is the most significant gap in the Results section. The manuscript groups interventions loosely under CBT, MBIs, and multi-component programmes without applying any recognised classification framework. In reality, the included studies span meaningfully distinct intervention types — including standalone CBT, standalone BA, MBCT, MBSR, ACT, DBT, psychodynamic therapy, psychoeducation, collaborative care models, stepped-care models, peer-facilitated interventions, and digital self-guided programmes. These differ substantially in their theoretical basis, active components, and delivery mechanisms, yet they are conflated under broad umbrella terms. The authors should apply a recognised taxonomy — such as the TIDieR framework dimensions or a comparable classification system — to organise and present the intervention landscape systematically.

9. A Dedicated Intervention Classification Table is Needed

Table 2 summarises included studies by design type but does not provide a structured classification of interventions. A dedicated table presenting, for each study or intervention type, the intervention name, theoretical orientation, active components, delivery mode, provider type, duration, and whether the intervention was adapted specifically for MLTCs or repurposed from single-condition contexts, would substantially improve the utility of this review for readers and align it with the scoping review's primary purpose of mapping the evidence.

10. The Multi-Component Intervention Figures Are Not Reconciled

The manuscript states that 81% of studies used multi-component interventions, yet separately reports CBT in 69% and MBIs in 31% of studies as though these are distinct categories. These figures are not reconciled, creating confusion about whether CBT and MBIs were predominantly standalone or components within broader programmes. The authors should present standalone and component uses of each modality separately.

11. Delivery Mode and Provider Type Are Not Mapped Quantitatively

Despite being described as implementation-relevant parameters, delivery mode and provider type are presented only through narrative prose and illustrative citations, with no systematic counts or proportions. Given the authors' stated emphasis on implementation gaps, these parameters should be quantified and presented in a structured format.

12. The Outcomes and Interventions Subsections are Inconsistent in Rigour

The mental health outcomes subsection presents counts, percentages, and named instruments in a structured manner. The interventions subsection does not achieve the same level of rigour. This imbalance suggests the intervention data were not subjected to equivalent systematic charting and should be corrected.

MINOR CONCERNS

13. Novelty Claim Requires Substantiation. The claim that this is the "first comprehensive scoping review" of this topic must be supported by documented evidence, or appropriately qualified.

14. Abstract Headings. The headings "Findings" and "Interpretation" should be revised to align with standard PLOS ONE abstract conventions (Background, Methods, Results, Conclusions).

15. Figure 2 Legend. "Africa" appears in the legend but has no corresponding slice in the pie chart. This should be corrected or explained.

16. Word Count. At 3,496 words, the manuscript is exceptionally brief for a scoping review of 54 studies. The thin methods and results reporting likely reflects this constraint, and the authors should consider whether the current length permits adequate reporting.

17. Typographical Errors. A missing full stop appears in the Methods section ("…wider research team The first author…"). The funding statement contains a spelling error ("Noth East Somerset" should read "North East Somerset").

Reviewer #2: Thank you for the opportunity to review this paper. The manuscript presents a scoping review of psychological interventions for mental health in adults living with multiple long-term conditions (MLTCs). The topic is timely and relevant given the increasing global burden of multimorbidity and its associated mental health challenges. The review is generally well-written, methodologically informed, and draws on a substantial and diverse body of literature.

Below are my comments/suggestions the authors may find useful for improving the overall quality of the paper.

Abstract

The abstract methods section is quite scanty and could be strengthened by providing additional methodological detail. The authors may briefly describe how studies were selected and screened, including the study selection process and any eligibility procedures used. It may also be useful to indicate the methodological framework or reporting guideline adopted for the scoping review (e.g., PRISMA-ScR), as well as the types of studies included

Introduction

Although the introduction repeatedly states that the evidence base is “fragmented,” “underdeveloped,” or “underexplored,” the specific knowledge gap remains somewhat broad. The authors may more clearly specify what is currently unknown, why existing evidence syntheses are insufficient, and how this scoping review uniquely advances the literature. For instance, it is unclear whether previous reviews excluded MLTC populations, focused only on specific psychological interventions, or failed to examine the evolution of the evidence base over time.

Methods

1. Although the authors indicate that the review followed Arksey and O’Malley’s framework and PRISMA-ScR guidelines, the eligibility criteria are not sufficiently described within the methods narrative. The authors should clearly specify the inclusion and exclusion criteria beyond referring readers to Table 1, including eligible study designs, publication types, language restrictions, and whether grey literature was considered.

2. The search strategy section could be improved by indicating whether reference list screening, citation tracking, hand-searching, or grey literature searches were conducted. Given the evolving nature of MLTC research, omission of these sources may limit comprehensiveness.

3. The data charting and synthesis process is described only briefly and requires further clarification. The authors should provide more detail on how data extraction/charting was conducted, whether the extraction process was performed independently by more than one reviewer, whether the extraction form was piloted or refined iteratively during the review process, and how disagreements arising during data extraction were resolved

Results

The categorization of evidence strands requires further clarification. The distinction between studies focusing on a specific LTC plus depression and those adopting broader MLTC definitions is useful, but the criteria used to classify studies into these strands are not fully explained.

Discussion

The “Comparison with previous literature” section appears somewhat selective and primarily confirmatory, with limited critical engagement. Although it links the review findings to existing studies, it does not sufficiently interrogate, contrast, or explain inconsistencies within the broader literature. In particular, the section would be strengthened by a more explicit discussion of areas where the current findings diverge from prior reviews, as well as a deeper exploration of why certain anticipated evidence domains—such as more robust implementation research or greater diversity in intervention approaches—remain underdeveloped or absent.

**********

what does this mean?). If published, this will include your full peer review and any attached files.

If you choose “no”, your identity will remain anonymous but your review may still be made public.

**Do you want your identity to be public for this peer review?**  For information about this choice, including consent withdrawal, please see our Privacy Policy

Reviewer #1: No

Reviewer #2: No

**********

[NOTE: If reviewer comments were submitted as an attachment file, they will be attached to this email and accessible via the submission site. Please log into your account, locate the manuscript record, and check for the action link "View Attachments". If this link does not appear, there are no attachment files.]

To ensure your figures meet our technical requirements, please review our figure guidelines: https://journals.plos.org/plosone/s/figures

You may also use PLOS’s free figure tool, NAAS, to help you prepare publication quality figures: https://journals.plos.org/plosone/s/figures#loc-tools-for-figure-preparation.

NAAS will assess whether your figures meet our technical requirements by comparing each figure against our figure specifications.

Attachment Submitted filename: Report.docx

# Author comment: Author response to Decision Letter 1

**DOI:** 10.1371/journal.pone.0355366.r002

17 Jun 2026

We are sincerely grateful to the reviewers for the time and effort spent reviewing our manuscript, titled “Psychological interventions for mental health in adults with multiple long-term conditions: A scoping review and evolution of the evidence base.” We have carefully addressed all comments below, point-by-point. Based on this feedback, we have (a) substantially expanded the Methods section to provide more detail on our process, (b) reworked the Results section to give only factual information, reserving interpretation for the Discussion, (c) restructured and expanded the Discussion to clarify how this review extends the evidence base, and (d) provided a new, detailed Table 3 giving data breakdown by study.

Reviewer 1

This manuscript addresses a timely and important public health topic. The use of the MRC Complex Interventions Framework to chart the evolution of the evidence base is a strength. However, several fundamental methodological concerns must be addressed before the manuscript can be considered for publication.

We thank Reviewer 1 for their detailed and clearly presented review of our manuscript. We appreciate your commendation of our work’s strengths and contribution to the literature. We made changes to the submission in line with your and Reviewer 2’s recommendations and believe that the paper is indeed clearer and stronger for it.

MAJOR CONCERNS

1. Inclusion of Systematic Reviews as Primary Data Sources

Seven systematic reviews and meta-analyses are included among the 54 studies and treated as equivalent to primary studies during data extraction. This is methodologically problematic for three reasons: it introduces a unit-of-analysis error by conflating aggregated and primary evidence; it risks double-counting primary studies that may have been captured both within those reviews and independently through the authors' own database searches; and it means findings are extracted from already-interpreted data, adding an additional layer of potential bias. The authors must either exclude systematic reviews from the evidence synthesis and use them only to inform the background and discussion, or provide explicit methodological justification for their inclusion with a clear description of how overlap with primary studies was managed. If reviews are retained as a distinct evidence category, this should be clearly labelled and handled as a separate strand of the synthesis.

Thank you for raising this point thoughtfully. The inclusion of review papers in the scoping review was intentional, since the aim was to map all the available evidence in the area, which mandates including reviews. To address your concern, we separated the review findings out from studies based on primary data and now summarise them in the “Review-level evidence” section appended below.

“Review-level findings

Seven systematic reviews met the inclusion criteria, all of which mapped onto Stage 3 (Effectiveness evaluation) of the MRC framework. These reviews mostly synthesised data across populations defined by a single physical long-term condition paired with a comorbid mental health issue. Specifically, the reviews focused on depression and/or anxiety co-occurring with chronic obstructive pulmonary disease (COPD);73 chronic musculoskeletal pain;70 generic chronic pain;74 and diabetes.75 Three reviews broader in scope evaluated dyads of chronic somatic conditions and anxiety/depression76,77 or focused purely on psychiatric co-occurring mood and anxiety disorders in older adults.78 The reviewed intervention modalities ranged from any psychological interventions in four reviews73,75,76,78 to a narrower scope of CBT-based interventions,74 remotely delivered interventions,70 or remotely-delivered CBT.77 While depression and anxiety were universally tracked as the primary outcomes, several reviews also captured impacts on stress, wellbeing, self-rated health, and condition-specific functional impacts.76,77

In terms of future work, review-level evidence recommended shifts toward policy and clinical practice changes to integrate these interventions into routine care pipelines. 70,73,76 Concurrently, authors underscored the need for primary evidence at the fourth MRC Stage (i.e., implementation research),70,76 alongside more rigorous effectiveness trials within their respective populations.74,75,78 Finally, review-level literature highlighted the necessity of evaluating diverse, socioeconomically varied cohorts within low- and middle-income countries.77”

To address the risk of double-counting you identify, we cross-checked the primary studies included in each of the seven reviews against the 48 primary studies in our own selection. This confirmed that five primary studies were captured both within a review and independently through our database searches (three of which derive from Sanabria-Mazo and colleagues' 2023 review). The overlap is therefore modest and does not materially affect the evidence map, but we now make it explicit in both the Methods and Limitations sections so that readers can clearly see how the two strands relate.

Addition to Method:

“Because review articles and primary studies were both eligible, we cross-checked the primary studies synthesised within each included review against those identified independently through our database searches. Five primary studies31-35 appeared in both strands; these were retained and reported in both the primary and review-level syntheses without adjustment, since the two strands are presented separately and serve different mapping purposes.”

Addition to Discussion:

“Relatedly, a small number of primary studies (five) were captured both independently and within the included reviews, three of which derived from a single recent review.75 This overlap was modest and, given that primary and review-level evidence are reported as separate strands, it does not substantially change the interpretation of the evidence map.”

2. Methods Section is Insufficiently Detailed

The Methods section is too brief for a scoping review. Several critical details are missing. The full search strategy is not presented in the manuscript body as required by PRISMA-ScR; relegating it entirely to supplementary files is insufficient. The rationale for the January 2000 start date is not explained. The data extraction process is not adequately described — it is unclear whether extraction was conducted by one or two reviewers, and no inter-rater reliability figure is provided for extraction (only for screening). The charting and synthesis process is described in one sentence and does not explain how data were coded or categorised against the five review questions. These omissions need to be addressed in full.

We agree with your assessment and have substantially expanded the Methods section, addressing all your points. To avoid copying the whole new section here, we invite you to read the additions highlighted in yellow in the revised manuscript.

3. Discrepancy Between the Abstract, Methods, and PRISMA Flow Diagram

The abstract and Methods section state that six databases were searched, but the PRISMA flow diagram lists eight sources, including ClinicalTrials.gov and ICTRP. This inconsistency must be resolved. If trial registries were searched, this should be stated in the Methods and the implications for screening described. If they were not, the flow diagram requires correction.

Thank you for your attention to detail in our reporting. We searched the stated six databases directly through their websites, however, the Cochrane Library database also includes ClinicalTrials.gov and WHO’s ICTRP records. The PRISMA diagram, which was auto-generated from the Covidence software, reports the original source (e.g., ICTRP), even if the record was identified through a Cochrane Library search. We now clarify this in the Method, as well as explain that we ultimately did not include trial registrations and grey literature as eligible studies.

4. Numerical Inconsistencies in Study Counts

The study design categories listed in the Results section sum to 55, not 54 as reported. This discrepancy must be identified and corrected across the manuscript, tables, and flow diagram.

The numerical discrepancy arose because one study (Sanabria-Mazo et al.’s IMPACT study) had a hybrid effectiveness-economic evaluation design, and was counted under both designs. We now clarify this in the breakdown of research designs. The figures were left with N = 54, since the total of studies remains the same.

5. Absence of Any Quality Consideration

While quality appraisal is not mandatory in scoping reviews, the authors draw direct clinical and policy recommendations from their findings. In this context, the complete absence of any quality consideration is difficult to justify. The authors should at minimum acknowledge this explicitly as a limitation and discuss how it affects confidence in their recommendations.

As per established methodological frameworks for scoping reviews, such as the Arksey and O’Malley (2005) framework we followed, formal quality assessment of included studies is not advised. Because the primary objective of a scoping review is to comprehensively map the extent, range, and nature of the existing evidence base rather than to synthesize effects to determine a definitive clinical answer, a formal quality appraisal was not undertaken. Our recommendations are largely based on the current split of available publications by types of condition, research designs, and geographical locations, to which quality assessment is less crucial. However, we did apply a minimal quality criterion during our selection process to safeguard the integrity of the charted data: we restricted our inclusion to peer-reviewed publications and excluded unreviewed student theses or conference abstracts. We added a brief statement to the Methods section to make this rationale clearer to the reader. We also now acknowledge this in the Discussion as a limitation:

“The lack of formal quality assessment, while in line with best-practice guidelines, means that our recommendations are based on all available evidence rather than prioritising the highest-quality outputs only.”

6. MRC Stage Classification is Not Explained

The assignment of studies to MRC stages is central to one of the paper's key contributions, yet no decision rules or criteria are described. Who made these assignments, on what basis, and were they checked independently? Without this, Figure 3 and the associated narrative cannot be evaluated.

The classification into MRC stages was made as follows: the foundational MRC Complex Interventions text (Skivington et al., 2021) splits the scientific process in the four reported phases (1. Intervention development/identification, 2. Feasibility/acceptability/pilot testing, 3. Effectiveness evaluation, 4. Implementation/economic evaluation). The first author matched the author-reported aims of each eligible study to these phases using the ‘select all that apply’ approach, since some studies reported on multiple phases (e.g., describing intervention development and feasibility trial in one publication)—where multiple categories applied, we noted all but counted the highest available stage in Figure 3, since this is most useful for interpreting the state of the evidence base. As with all our data extraction and interpretation, any ambiguities were resolved in discussion with multiple authors. We added a statement to the Limitations section to acknowledge the fact that our data extraction (which this scoring was part of) was not independently done by two authors in full:

“Our title/abstract screening and data extraction were not done in full by two independent scorers; instead, the process was piloted on 10% of the papers at either stage, then one author completed the work and highlighted any ambiguous records to discuss with the full team. While this process was efficient and maintained the strength of multiple perspectives, it lacks the rigour of independent double screening and extraction.”

CONCERNS ABOUT THE RESULTS SECTION

7. Results Read as a Narrative Review Rather Than a Scoping Review

The Results section presents findings through interpretive prose rather than structured mapping, which is more characteristic of a narrative review. Statements describing "emerging trends" and intervention emphases reflect the authors' synthesised reading of the literature and belong in the Discussion. The Results section should present a transparent, quantified account of what was found, reserving interpretation for later.

Thank you for the methodological guidance. We agree that the highlighted wording (“emerging trend”) is better placed in the discussion, so we revised the results section to read as factual. We also moved the “Evolution of evidence base” section into the Discussion, since it required a degree of author interpretation. We feel that with this revised wording, a narrative format is still most suitable for a coherent overview of the current literature, and is in line with the methodological framework we followed (Arksey & O’Malley, 2005), as well as most published scoping reviews. We also clarify that a narrative synthesis approach was used in the Method, since this was most suitable for meeting the review’s range of aims:

“A narrative synthesis was deemed the most suitable to convey our results (Popay et al., 2006).”

8. Interventions Are Not Classified Using a Recognised Framework

This is the most significant gap in the Results section. The manuscript groups interventions loosely under CBT, MBIs, and multi-component programmes without applying any recognised classification framework. In reality, the included studies span meaningfully distinct intervention types — including standalone CBT, standalone BA, MBCT, MBSR, ACT, DBT, psychodynamic therapy, psychoeducation, collaborative care models, stepped-care models, peer-facilitated interventions, and digital self-guided programmes. These differ substantially in their theoretical basis, active components, and delivery mechanisms, yet they are conflated under broad umbrella terms. The authors should apply a recognised taxonomy — such as the TIDieR framework dimensions or a comparable classification system — to organise and present the intervention landscape systematically.

We thank the reviewer for raising an important point regarding intervention classification. Our approach to grouping the interventions was directly guided by, and limited by, the level of detail provided in the primary papers. To avoid over-interpretation or mischaracterisation without a sufficient level of granular data, we chose to classify the interventions based on the terms and descriptions explicitly used by the primary authors themselves.

We completely agree that the TIDieR framework is an excellent and valuable tool for structuring intervention reporting; however, it prescribes how an intervention's components should be described (e.g., location, frequency, provider) rather than offering a standardised taxonomic framework for categorising psychological approaches beyond the groupings we adopted. We also share the reviewer's view that reporting specific mechanisms of action and discrete therapeutic techniques would have added significant depth to the review. Nonetheless, because the necessary depth of reporting was absent in the majority of the included primary papers, applying a highly granular classification system (e.g., Michie et al.’s Behaviour Change Ontology) was beyond the scope of this scoping review. In light of this, we added a future direction for research (“Report complex interventions thoroughly, ideally following a standardised classification framework (e.g., Behaviour Change Taxonomy1 or Ontology1) to aid study replication and evidence synthesis.”) and a reflection on this reporting limitation to our Discussion section:

“We mapped intervention approaches into broad categories (CBT, MBIs, BA, psychoeducation, and integrated care), without following a structured classification system. Our approach to grouping the interventions was directly guided by, and limited by, the level of detail provided in the primary papers. To avoid over-interpretation or mischaracterisation, we chose to classify the interventions based on the terms and descriptions used by the primary authors themselves. A future direction—dependent on sufficiently detailed reporting—would be to map evidence and its hypothesised mechanisms of action according to granular intervention classification systems (e.g., The Behaviour Change Ontology), which was presently not possible.”

9. A Dedicated Intervention Classification Table is Needed

Table 2 summarises included studies by design type but does not provide a structured classification of interventions. A dedicated table presenting, for each study or intervention type, the intervention name, theoretical orientation, active components, delivery mode, provider type, duration, and whether the intervention was adapted specifically for MLTCs or repurposed from single-condition contexts, would substantially improve the utility of this review for readers and align it with the scoping review's primary purpose of mapping the evidence.

Thank you for your suggestion. We have now replaced Table 2 with a more comprehensive table (now Table 3) giving a breakdown by study instead of a summary. Together with the revised text summary of the evidence base, this gives the most efficient overview according to the needs of the reader.

10. The Multi-Component Intervention Figures Are Not Reconciled

The manuscript states that 81% of studies used multi-component interventions, yet separately reports CBT in 69% and MBIs in 31% of studies as though these are distinct categories. These figures are not reconciled, creating confusion about whether CBT and MBIs were predominantly standalone or components within broader programmes. The authors should present standalone and component uses of each modality separately.

We now specify the prevalence of each approach individually and emphasise the large proportion of intervention reporting multiple modalities, such as: “Studies evaluated a range of psychological intervention approaches. The most frequent approaches were CBT (62% of studies) and BA (70%), either as a standalone intervention (CBT in 4%49,50 and BA in 4%39,40) or a core component in multi-modal programmes.”

Please note that the percentages have changed slightly from the initial submission because we removed the systematic reviews from this section of the results.

11. Delivery Mode and Provider Type Are Not Mapped Quantitatively

Despite being described as implementation-relevant parameters, delivery mode and provider type are presented only through narrative prose and illustrative citations, with no systematic counts or proportions. Given the authors' stated emphasis on implementation gaps, these parameters should be quantified and presented in a structured format.

Thank you for the suggestion. We now quantify the different categories of delivery mode and provider type as requested:

“Modes of delivery were split between in-person (51%), remote (i.e., telephone, video-conferencing, or digital platforms; 26%), and mixed delivery (21%). Interventions were delivered by dedicated study staff (26%),32,39,48,65 primary care providers or nurses (19%),41,59,66,67 other healthcare professionals (e.g., psychologists or PwPs; 62%),30,33,38,42,51 or self-delivered (9%)44,46,49,68 alone or in combination—in total, just under a quarter involved multiple delivery providers.37,60,66,69 A handful of studies reported using innovative facilitators, such as community pharmacists,40 osteopaths,58 or mindfulness teachers.55.”

12. The Outcomes and Interventions Subsections are Inconsistent in Rigour

The mental health outcomes subsection presents counts, percentages, and named instruments in a structured manner. The interventions subsection does not achieve the same level of rigour. This imbalance suggests the intervention data were not subjected to equivalent systematic charting and should be corrected.

We thank you for this observation and appreciate the opportunity to clarify. The mental health outcomes section allowed for a higher degree of numerical precision because tracking psychiatric symptom domains and instruments is highly structured by nature (e.g., a diagnostic tool either evaluates depression or it does not), reflecting the core psychiatric focus of this scoping review. In contrast, the psychological interventions evaluated across the primary literature were highly heterogeneous, with unique and complex designs, varied delivery protocols, and frequently omitted or inconsistently reported data regarding specific durations and active therapeutic elements. Attempting to force these narrative and often incomplete descriptions into rigid percentages or homogenous categories would have required an inappropriate degree of author interpretation and risked over-generalising complex interventions. To maintain methodological rigour without misrepresenting the primary data, we chose to present the high-level thematic patterns within the text while providing the granular specifics for each intervention in revised Table 2. We believe this layout is most useful to the widest array of readers.

MINOR CONCERNS

13. Novelty Claim Requires Substantiation. The claim that this is the "first comprehensive scoping review" of this topic must be supported by documented evidence, or appropriately qualified.

In response to your and Reviewer 2’s suggestion, we removed the ‘first comprehensive scoping review’ statement, clarified the challenges in existing literature, and specified how this review addresses them. The section now reads:

“Research on MLTCs is in relatively early stages and facing several challenges. Firstly, a historical focus on single conditions in research and clinical practice has led to a legacy of assuming that single-condition insights are merely additive.16 This perspective overlooks the complexity of living with MLTCs, where interactions between conditions’ symptoms, effects and treatments can lead to unexpected outcomes or an added burden. Secondly, the evidence base for interventions specifically targeting individuals with MLTCs is underdeveloped considering its rising prevalence and impact,2 reflecting the relatively recent recognition of the complexity it entails (both experientially and in terms of treatment needs)10,17 and uptick in dedicated research funding.18,19 Thirdly, the literature is fragmented into groupings of conditions based on incidental or funding-mandated foci (e.g., funding calls for interventions targeting a specific class of conditions),20 making a coherent overview difficult. Finally, the accumulation of knowledge on MLTC treatment is further complicated by variation in the definition of a long-term health condition (i.e., whether to include only physical conditions or also chronic mental health conditions).21 In this review, we adhere to the UK Academy of Medical Sciences’ definition, where each of the co-occurring conditions is either i) a physical non-communicable disease of long duration, ii) a mental health condition of long duration, or iii) an infectious disease of long duration.21 As a result of these challenges, existing reviews synthesise intervention literature for specific combinations of conditions (such as depression and cardiovascular disease),22 focus on a single approach for groups with MLTCs (e.g., mindfulness-based interventions),23 expand their focus to all complex interventions,24 only consider physical LTCs in their scope,25 or only review one type of research design (e.g., a qualitative synthesis).10 No work to date has followed the increasingly endorsed AMS/NIHR definition of MLTCs, mapped out the evidence landscape across psychological approaches, and charted its evolution over time.”

14. Abstract Headings. The headings "Findings" and "Interpretation" should be revised to align with standard PLOS ONE abstract conventions (Background, Methods, Results, Conclusions).

Abstract was edited as suggested to bring it in line with PLOS One formatting.

15. Figure 2 Legend. "Africa" appears in the legend but has no corresponding slice in the pie chart. This should be corrected or explained.

Indeed—there is currently no work with first author’s affiliations based in Africa, meaning that the category has no corresponding slice in the chart. We deem it important to include all continents in the legend, since this helps emphasise our point for geographical imbalance of the current evidence base. We now explain the discrepancy as a note under Figure 2:

“Note: Location determined by the country of the first author’s institution. In the case of multiple institutions, the primary institution was used. There are currently no publications with primary author publications based in Africa, therefore the category has no corresponding section on the chart.”

16. Word Count. At 3,496 words, the manuscript is exceptionally brief for a scoping review of 54 studies. The thin methods and results reporting likely reflects this constraint, and the authors should consider whether the current length permits adequate reporting.

We appreciate the Reviewer’s reflection on the brevity of our report. The length was due to formatting restrictions suggested by some of the leading publications in the field (e.g., The Lancet Group), which emphasise concise publications. With the revisions and expanded Methods/Results/Discussion sections, the manuscript now stands at 5143 words.

17. Typographical Errors. A missing full stop appears in the Methods section ("…wider research team The first author…"). The funding statement contains a spelling error ("Noth East Somerset" should read "North East Somerset").

Many thanks—the identified typos have been corrected.

Reviewer 2

Thank you for the opportunity to review this paper. The manuscript presents a scoping review of psychological interventions for mental health in adults living with multiple long-term conditions (MLTCs). The topic is timely and relevant given the increasing global burden of multimorbidity and its associated mental health challenges. The review is generally well-written, methodologically informed, and draws on a substantial and diverse body of literature.

Below are my comments/suggestions the authors may find useful for improving the overall quality of the paper.

We appreciate Reviewer 2’s thoughtful review and suggestions. We carefully considered an addressed each point in turn below.

Abstract

The abstract methods section is quite scanty and could be strengthened by providing additional methodological detail. The authors may briefly describe how studies were selected and screened, including the study selection process and any eligibility procedures used. It may also be useful to indicate the methodological framework or reporting guideline adopted for the scoping review (e.g., PRISMA-ScR), as well as the types of studies included.

Thank you. We expanded on our methodology and reporting frameworks, and provided more detail on the search parameters, as much as the Abstract word count allows. Its Methods section now reads:

“Methods: The pre-registered review follows Arksey & O’Malley’s (2005) framework and is reported in line with the PRISMA-ScR guidelines. We searched six databases (PubMed, Embase, Scopus, Cochrane, PsycNET, Web of Science) up to January 2025, including reference screening and a grey literature search. After screening 124 full texts, 54 studies were included. Eligible studies evaluated any psychological intervention in adults with two or more long-term health conditions and included a validated psychological health outcome.”

Introduction

Although the introduction repeatedly states that the evidence base is “fragmented,” “underdeveloped,” or “underexplored,” the specific knowledge gap remains somewhat broad. The authors may more clearly specify what is currently unknown, why existing evidence syntheses are insufficient, and how this scoping review uniquely advances the literature. For instance, it is unclear whether previous reviews excluded MLTC populations, focused only on specific psychological interventions, or failed to examine the evolution of the evidence base over time.

We reworked and expanded the paragraph characterising the current MLTC literature, including existing reviews. We also specify the contribution of this review before giving its aims:

“Research on MLTCs is in relatively early stages and facing several challenges. Firstly, a historical focus on single conditions in research and clinical practice has led to a legacy of assuming that single-condition insights are merely additive.16 This perspective overlooks the complexity of living with MLTCs, where interactions between conditions’ symptoms, effects and treatments can lead to unexpected outcomes or an added burden. Secondly, the evidence base for interventions specifically targeting individuals with MLTCs is underdeveloped considering its rising prevalence and impact,2 reflecting the relatively recent recognition of the complexity it entails (both experientially and in terms of treatment needs)10,17 and uptick in dedicated research funding.18,19 Thirdly, the literature is fragmented into groupings of conditions based on incidental or funding-mandated foci (e.g., funding calls for interventions targeting a specific class of conditions),20 making a coherent overview difficult. Finally, the accumulation of knowledge on MLTC treatment is further complicated by variation in the definition of a long-term health condition (i.e., whether to include only physical conditions or also chronic mental health conditions).21 In this review, we adhere to the UK Academy of Medical Sciences’ definition, where each of the co-occurring conditions is either i) a physical non-communicable disease of long duration, ii) a mental health condition of long duration, or iii) an infectious disease of long duration.21 As a result of these challenges, existing reviews synthesise intervention literature for specific combinations of conditions (such as depression and cardiovascular disease),22 focus on a single approach for groups with MLTCs (e.g., mindfulness-based interventions),23 expand their focus to all complex interventions,24 only consider physical LTCs in their scope,25 or only review one type of research design (e.g., a qualitative synthesis).10 No work to date has followed the increasingly endorsed AMS/NIHR definition of MLTCs, mapped out the evidence landscape across psychological approaches, and charted its evolution over time.”

Methods

We agree with the reviewer’s assessments and have substantially expanded the Methods section, addressing your points 1-3 below. Please see below for each point in turn, copied from the revised manuscript.

1. Although the authors indicate that the review followed Arksey and O’Malley’s framework and PRISMA-ScR guidelines, the eligibility criteria are not sufficiently described within the methods narrative. The authors should clearly specify the inclusion and exclusion criteria beyond referring readers to Table 1, including eligible study designs, publication types, language restrictions, and whether grey literature was considered.

We now summarise the eligibility criteria in text (as well as pointing to full criteria in Table 2). It now reads:

“We searched six databases (PubMed, Embase, Scopus, PsycNET, Web of Science, Cochrane; the latter also includes ClinicalTrials.gov and ICTRP) for records including keywords related to psychological interventions, long-term conditions, and multimorbidity (see Table 1 for the search strategy). A search of reference lists, grey literature and hand search complemented the initial pool, although no eligible records were identified this way. To keep the review focused on quality-assessed work and capture the recent or current study of MLTCs, eligible studies had to be peer-reviewed papers published between January 2000 and January 2025 in English. After removal of duplicates, the first 10% of all titles and abstracts were independently screened by the first and senior authors against pre-specified eligibility criteria (see Table 2). In brief, eligible records reported on psychological interventions (defined as ≥50% of intervention time if the content was multi-modal) for adult samples with at least two LTCs (where a formal diagnosis of mental health conditions was not needed but a diagnostic cut-off on a validated measure was), and measured at least one validated mental health outcome. We included empirical research with primary or secondary data, but excluded conference abstracts, trial registrations, unreviewed theses, and commentaries. Review articles were deemed important to include as signals of interest and/or higher quantity in a particular area of MLTC interventions. Similarly, peer-reviewed protocols were included as an indicator of research to come—both of which aided our mapping of the literature’s evolution.”

2. The search strategy section could be improved by indicating whether reference list screening, citation tracking, hand-searching, or grey literature searches were conducted. Given the evolving nature of MLTC research, omission of these sources may limit comprehensiveness.

We now clarify that we conduced reference screening and a grey literature search, although not citation tracking. See the text copied above (second sentence).

3. The data charting and synthesis process is described only briefly and requires further clarification. The authors should provide more detail on how data extraction/charting was conducted, whether the extraction process was performed independently by more than one reviewer, whether the extraction form was piloted or refined iteratively during the review process, and how disagreements arising during data extraction were resolved.

Thank you. This has been done, and now reads:

“Data extraction recorded the general study information (e.g., year of publication, country of first author), study design, MRC Framework stage26 (1. Intervention development/identification, 2. Feasibility/acceptability/pilot testing, 3. Effectiveness evaluation, 4. Implementation/economic evaluation; if a study satisfied the criteria for multiple stages, such as a hybrid effectiveness-implementation design, it was counted towards all categories), participant conditions and how they were determined (Self-report, Diagnostic assessment by clinician, Other), number of participants, type of intervention that applied (CBT, ACT, Mindfulness, DBT, BA, Psycho-educational, Integrated/stepped care, Other), the setting (Community, Educational, Primary care, Healthcare other than primary care, Other), delivery mode (In person, Digital/remote, Mixed), facilitator (Primary care practitioner, Other healthcare practitioner (e.g., PwP), Dedicated study staff, Carer, Self-delivered, Other), duration, frequency, and descriptions of intervention content and any control conditions (as reported by authors). In terms of outcomes, we extracted the outcome domain (Depression, Anxiety, Wellbeing, Stress, Self-rated health, Condition-specific impact on mental health (e.g., diabetes distress), Other), validated measures used, how they were assessed (Self-report questionnaire, Assessment by clinician, Other medical procedure) and at what timepoints, and which groups were assessed (Patients, Carers/family members, Healthcare providers, Policymakers, Other). We recorded the direction of effect on mental health (Improved, Mixed, No change, Deteriorated), a summary of qualitative findings (as applicable), study limitations, funding sources and conflicts of interest, and any other notes. The extraction template was prepared in advance and piloted in the research team, which resulted in addition of fields recording the National Health Service Quality and Outcomes Framework condition groups (NHS QOF; i.e., categorising as Cardiovascular, Respiratory, Musculoskeletal, Mental health and Neurology, Other LTCs (e.g., cancer, kidney disease))29 on top of author-listed LTCs, and recommendations the extracted study makes for future work (categorised as None specified, Cease research, Further research at this stage, Next stage research, Policy/practice change, Other). Data extraction was completed by the first author, who has extensive experience with literature synthesis research, in consultation with the senior author where data were ambiguous. The full search strategy, extraction template and data are available in Supplementary materials.

Data synthesis involved organising the data in line with our review questions: 1) when, where and by whom the research was done, 2) which conditions did they include as LTCs, 3) which types of psychological interventions did they study, 4) which mental health outcomes were targeted, and 5) what recommendations did the articles include for research, practice, and policy. Our interpretation was an iterative process led by the first author with input from the whole research team, guided by a summary spreadsheet with sections mapped on the review questions, and colour-coded by MRC Stage of Evidence, research design, and geographical location. The synthesis and interpretation presented in the following sections are our attempt to answer the review aims (i.e., what evidence exists on psychological interventions for adults with MLTCs, how it is evolving over time, and what it presently can(not) tell us). In line with best practice guidelines for scoping review methodology,27 we did not undertake formal statistical synthesis nor assess risk of bias, although only considering peer-reviewed publications from indexed journals served as a minimal quality threshold.”

Results

The categorization of evidence strands requires further clarification. The distinction between studies focusing on a specific LTC plus depression and those adopting broader MLTC definitions is useful, but the criteria used to classify studies into these strands are not fully explained.

We classified the research based on authors’ reporting of the aims, target population and content of the interventions. In most cases of strand 1 research (single LTC + co-morbid depression), the authors pre-specified which single LTC was targeted, clarified that the sample was presenting with co-morbid depression (i.e., arising simultaneously and contingent on the primary LTC), and frequently tailored their treatment to the specific combination. For example, Tovote et al (2014; https://pubmed.ncbi.nlm.nih.gov/24898301/) tested an MBCT intervention specifically tailored to patients with diabetes who were experiencing high depression symptoms. In strand 2, authors reported more flexibility in their recruitment (e.g., any two physical LTCs) and interventions included content tailored to the complexity of multimorbidity. The two strands have different implications for advancing the literature on MLTC management, which is why we comment on the distinction in our Results.

Discussion

The “Comparison with previous literature” section appears somewhat selective and primarily confirmatory, with limited critical engagement. Although it links the review findings to existing studies, it does not sufficiently interrogate, contrast, or explain inconsistencies within the broader literature. In particular, the section would be strengthened by a more explicit discussion of areas where the current findings diverge from prior reviews, as well as a deeper exploration of why certain anticipated evidence domains—such as more robust implementation research or greater diversity in intervention approaches—remain underdeveloped or absent.

Thank you for your suggestion. We reorganised and expanded the Discussion section, which now more critically evaluates the state of the current literature, including a paragraph clarifying the contributions of this review (leading up to the Recommendations for research, policy and practice section):

“Nevertheless, this review both builds on and challenges existing research. First, it highlights the increasing importance of integrated healthcare, with multidisciplinary teams and shared-care approaches. Our mapping captured the promise of intervention delivery within unexpected settings and by non-traditional providers—such as community pharmacists40 and osteopaths58—illustrating a shift toward pragmatic, embedded physical-mental healthcare models that prior reviews have largely overlooked. Secondly, our broad criteria for eligible designs and intervention types highlights the varied array of approaches being considered for mental health management in groups with MLTCs. Given the burden and growing prevalence of this public health issue, collating all these attempts in one place—from published case studies to forthcoming economic evaluations—allows a helpful overview of the topic. Lastly, while earlier reviews primarily focused on establishing clinical efficacy of interventions, mapping the wider evidence base against the MRC framework allows us to highlight gaps across it, including the reasons for their existence. Our review demonstrates that the field is not stalling due to a lack of primary effectiveness data (Stage 3), but rather due to a young and developing evidence base focused specifically on multimorbidity as a condition unto itself, paired with absence of structural support for implementation and dissemination research (Stage 4) and health economic evaluations.”

Journal requirements

When submitting your revision, we need you to address these additional requirements.

1. Please ensure that your manuscript meets PLOS ONE's style requirements, including those for file naming. The PLOS ONE style templates can be found at

https://journals.plos.org/plosone/s/file?id=wjVg/PLOSOne_formatting_sample_main_body.pdf and

https://journals.plos.org/plosone/s/file?id=ba62/PLOSOne_formatting_sample_title_authors_affiliations.pdf

Thank you – this has been done.

2. Please include a separate caption for each figure in your manuscript.

Thank you – this has been done.

3. Please include your tables as part of your main manuscript and remove the individual files. Please note that supplementary tables should remain as separate "supporting information" files.

Thank you – this has been done, except for Table 3, which is too large to display in a Word document. Please see attached file “Table3.xlsx”.

4. Please include captions for your Supporting Information files at the end of your manuscript, and update any in-text citations to match accordingly. Please see our Supporting Information guidelines for more information: http://journals.plos.org/plosone/s/supporting-information.

Thank you – this has been done.

Attachment Submitted filename: Response to Reviewers.docx

# Peer review: Decision Letter 1

**DOI:** 10.1371/journal.pone.0355366.r003

**Authors:** Dorothy Serwaa Boakye

22 Jul 2026

Psychological interventions for mental health in adults with multiple long-term conditions: A scoping review and evolution of the evidence base

PONE-D-26-18140R1

Dear Dr. Lambert

We’re pleased to inform you that your manuscript has been judged scientifically suitable for publication and will be formally accepted for publication once it meets all outstanding technical requirements.

Within one week, you’ll receive an e-mail detailing the required amendments. When these have been addressed, you’ll receive a formal acceptance letter and your manuscript will be scheduled for publication.

An invoice will be generated when your article is formally accepted. Please note, if your institution has a publishing partnership with PLOS and your article meets the relevant criteria, all or part of your publication costs will be covered. Please make sure your user information is up-to-date by logging into Editorial Manager at Editorial Manager® and clicking the ‘Update My Information' link at the top of the page. For questions related to billing, please contact billing support.

If your institution or institutions have a press office, please notify them about your upcoming paper to help maximize its impact. If they’ll be preparing press materials, please inform our press team as soon as possible -- no later than 48 hours after receiving the formal acceptance. Your manuscript will remain under strict press embargo until 2 pm Eastern Time on the date of publication. For more information, please contact onepress@plos.org.

Kind regards,

Dorothy Serwaa Boakye, PGDE,MA,Mphil, RN, BSc

Academic Editor

PLOS One

Additional Editor Comments (optional):

Reviewers' comments:

Reviewer's Responses to Questions

**Comments to the Author**

Reviewer #1: All comments have been addressed

Reviewer #2: All comments have been addressed

**********

2. Is the manuscript technically sound, and do the data support the conclusions?

Reviewer #1: Yes

Reviewer #2: (No Response)

**********

3. Has the statistical analysis been performed appropriately and rigorously?

Reviewer #1: N/A

Reviewer #2: (No Response)

**********

4. Have the authors made all data underlying the findings in their manuscript fully available?

The PLOS Data policy

Reviewer #1: Yes

Reviewer #2: (No Response)

**********

5. Is the manuscript presented in an intelligible fashion and written in standard English?

Reviewer #1: Yes

Reviewer #2: (No Response)

**********

Reviewer #1: All reviewer number one comments have been substantially addressed in the manuscript. I recommend acceptance of this manuscript for publication.

Reviewer #2: (No Response)

**********

what does this mean?). If published, this will include your full peer review and any attached files.

If you choose “no”, your identity will remain anonymous but your review may still be made public.

**Do you want your identity to be public for this peer review?**  For information about this choice, including consent withdrawal, please see our Privacy Policy

Reviewer #1: No

Reviewer #2: No

**********

# Editor report: Acceptance letter

**DOI:** 10.1371/journal.pone.0355366.r004

**Authors:** Dorothy Serwaa Boakye

PONE-D-26-18140R1

PLOS One

Dear Dr. Lambert,

I'm pleased to inform you that your manuscript has been deemed suitable for publication in PLOS One. Congratulations! Your manuscript is now being handed over to our production team.

At this stage, our production department will prepare your paper for publication. This includes ensuring the following:

* All references, tables, and figures are properly cited

* All relevant supporting information is included in the manuscript submission,

* There are no issues that prevent the paper from being properly typeset

You will receive further instructions from the production team, including instructions on how to review your proof when it is ready. Please keep in mind that we are working through a large volume of accepted articles, so please give us a few days to review your paper and let you know the next and final steps.

Lastly, if your institution or institutions have a press office, please let them know about your upcoming paper now to help maximize its impact. If they'll be preparing press materials, please inform our press team within the next 48 hours. Your manuscript will remain under strict press embargo until 2 pm Eastern Time on the date of publication. For more information, please contact onepress@plos.org.

You will receive an invoice from PLOS for your publication fee after your manuscript has reached the completed accept phase. If you receive an email requesting payment before acceptance or for any other service, this may be a phishing scheme. Learn how to identify phishing emails and protect your accounts at https://explore.plos.org/phishing.

If we can help with anything else, please email us at customercare@plos.org.

Thank you for submitting your work to PLOS One and supporting open access.

Kind regards,

PLOS One Editorial Office Staff

on behalf of

Dr. Dorothy Serwaa Boakye

Academic Editor

PLOS One
